# Improvements in Patient‐Reported Outcomes and Few Reported Major Complications Following Hip Arthroscopy in Patients With Femoroacetabular Impingement Syndrome: A Systematic Review

**DOI:** 10.1002/ars2.70036

**Published:** 2026-06-08

**Authors:** Frederik Nicolai Foldager, Julius David, Lisa Urup Tønning, Bjarne Mygind‐Klavsen, Christian Gatzka, Barbara Zimmerman, Inger Mechlenburg

**Affiliations:** ^1^ Department of Orthopaedic Surgery Aarhus University Hospital Aarhus Denmark; ^2^ Department of Clinical Medicine Aarhus University Aarhus Denmark; ^3^ Hochschule 21 Buxtehude Germany; ^4^ Hamburg Hip and Knee Manufacture Hamburg Germany; ^5^ Exercise Biology Department of Public Health Aarhus University Aarhus Denmark

## Abstract

**Purpose:**

To systematically review the literature to evaluate complications and patient‐reported outcome measures (PROMs) after hip arthroscopy in patients with femoroacetabular impingement syndrome.

**Methods:**

Five databases were searched for studies reporting complications and PROMs after hip arthroscopy in patients with femoroacetabular impingement syndrome, excluding those with dysplasia or severe osteoarthritis. Complications included conversion to total hip arthroplasty, revision surgery, and nerve paresthesia. PROMs were assessed as mean changes from baseline in domains (pain, symptoms, activities of daily living, sports participation, quality of life, and composite scores). Descriptive subgroup analyses were performed by follow‐up duration and capsular repair. Risk of bias was assessed using the Cochrane Risk of Bias in Non‐randomized Studies of Interventions tool, and the certainty of the evidence was assessed using the Grading of Recommendations, Assessment, Development, and Evaluation approach.

**Results:**

Forty‐two studies (5885 hips) were included. The unweighted proportions of complications were 2.1% for conversion to total hip arthroplasty (range, 0%‐16.7%), 5.2% for revision surgery (range, 0%‐22.3%), and 3.8% for nerve paresthesia (range, 0.8%‐12.5%). PROMs (0‐100 scale; higher scores indicate greater improvement) showed mean changes from baseline to follow‐up ranging from 9.4 to 35.0 points for pain, 14.3 to 25.0 points for symptoms, 10.9 to 43.0 points for activities of daily living, 15.0 to 45.5 points for sports participation, 15.0 to 43.2 points for quality of life, and 10.3 to 47.6 points for composite scores. Evidence certainty was rated very low for complications and PROMs.

**Conclusions:**

Hip arthroscopy for femoroacetabular impingement syndrome is associated with low average rates of total hip arthroplasty conversion, revision surgery, and nerve paresthesia and with clinically meaningful improvements across PROM domains. However, the certainty of evidence is very low, and results should be interpreted with caution.

**Level of Evidence:**

Level IV, systematic review and subjective synthesis of Level I to IV studies.

## Graphical abstract

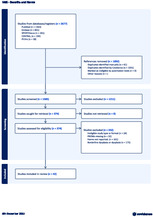

Femoroacetabular impingement syndrome (FAIS) is a symptomatic hip condition characterized by activity‐related hip or groin pain, functional limitations, and specific clinical and radiographic criteria.^1^ Although cam and pincer morphologies are relatively common in the general population,^2^, ^3^, ^4^ only a subset of individuals develop symptomatic FAIS, which is associated with pain, reduced hip function, and an increased risk of early osteoarthritis.^1^, ^5^, ^6^ Given the clinical burden of FAIS and its association with early‐onset hip osteoarthritis, surgical interventions have become increasingly utilized to address the underlying pathomechanics.

Hip arthroscopy is the most commonly used surgical intervention for FAIS. It is performed using traction and fluid infusion to access the joint. It includes, but is not limited to, femoroplasty, acetabuloplasty, labral repair or reconstruction, cartilage restoration techniques, and interventions targeting the ligamentum teres. Capsular repair or plication is often added to restore joint stability.^7^

Previous systematic reviews have reported complications associated with hip arthroscopy. However, many of these reviews included mixed patient populations, such as individuals with FAIS, dysplasia, borderline dysplasia, or severe osteoarthritis, which introduces a risk of selection bias because outcomes may differ substantially between diagnostic groups.^8^, ^9^, ^10^, ^11^, ^12^ Furthermore, studies without clearly defined prospective data collection may underestimate the frequency of complications, contributing to reporting bias.^13^ Prior studies have also relied on patient‐reported outcome measures (PROMs) that were not validated for use in patients with FAIS, limiting the ability to evaluate postoperative changes in symptoms and function accurately.^14^, ^15^

To address these limitations, we conducted a systematic review restricted to studies with prospectively collected data in patients with FAIS without dysplasia, borderline dysplasia, or severe osteoarthritis. The purpose of this study was to systematically review the literature to evaluate complications and PROMs after hip arthroscopy in patients with FAIS. We hypothesized that hip arthroscopy for FAIS would be associated with improvements in validated hip‐specific PROMs, whereas major complications would be infrequent.

## METHODS

### Search Strategy and Eligibility Criteria

This systematic review and subjective synthesis were conducted in accordance with the Cochrane Handbook for Systematic Reviews of Interventions and reported in accordance with the Preferred Reporting Items for Systematic Reviews and Meta‐Analyses guidelines.^16^ It was registered in the PROSPERO database on January 18, 2025 (CRD42025634200).

A systematic literature search was conducted on October 11, 2024, across 5 databases (MEDLINE, Embase, SPORTDiscus, Cochrane CENTRAL, and PEDro) with assistance from an experienced medical librarian from Aarhus University. The search combined terms related to FAIS, hip arthroscopy, and prospective study designs and was restricted to human studies published since 2005. All records were imported into Covidence (Covidence, Veritas Health Innovation, Melbourne, Australia) for screening by 2 independent reviewers (J.D., F.N.F.). Discrepancies were resolved through discussion and consensus. The full electronic search strategies for all databases are provided in Tables S1‐S5.

**TABLE 1:** Study Characteristics of the Included Studies

Author, Publication Year	Country	Study Design (LOE)†	Follow‐up Timepoints, Mean (SD)	Patients (hips)	Female, n (%)*	FAIS Morphology			Age, mean (SD)	BMI, mean (SD)
						Cam, n (%)*	Pincer, n (%)*	Mixed, n (%)*		
Summary, across all studies			41.2 (17.6) mo	5646 (5885)	3048 (54%)	1879 (32%)	639 (11%)	3119 (55%)	34.5 (11.7)	24.3(3.2)
Bech et al., 2021^32^	Netherlands	RCT (I)	3 mo and 1 yr	58 (58)	60	38	60	2	35.5 (10.4)	23.1 (2.7)
Bech et al., 2021^32^	Netherlands	RCT (I)	3 mo and 1 yr	58 (58)	67	39	61	0	33.5 (8.5)	24.2 (2.9)
Mullins et al., 2023^33^	Ireland	Cohort (III)	5 yr	120(120)	7	0	0	100	29 (8.7)	NA
Maldonado et al., 2022^34^	USA	Cohort (III)	31.4 mo	66 (66)	65	0	0	100	32.3 (12.2)	28.3 (6.4)
Fischer et al., 2024^35^	Germany	RCT (II)	6 mo	67 (67)	39	27	12	61	37.5 (NA)	25.5 (NA)
Honda et al., 2020^36^	Japan	Matched cohort (III)	31.3 mo	57 (57)	46	74	9	16	30.9 (10.9)	22.1 (3.1)
Honda et al., 2020^36^	Japan	Matched cohort (III)	31.3 mo	27 (27)	56	59	15	26	62.3 (3.4)	22.9 (2.8)
Waterman et al., 2018^37^	USA	Cohort (IV)	2 yr	29 (29)	21	86	14	0	36 (11.2)	25.2 (2.4)
Mullins et al., 2024^38^	Ireland	Cohort (IV)	10 yr	90 (90)	13	0	0	100	31.8(10)	NA
Moon et al., 2020^39^	South Korea	Cohort (IV)	5 yr	73 (90)	17	51	8	41	34.4 (NA)	24.1 (4.7)
Nawabi et al., 2016^40^	USA	Cohort (III)	5 yr	131(152)	56	15	0	85	29.6 (10.3)	NA
Dantas et al., 2021^41^	Portugal	Cohort (IV)	45 mo	154(160)	55	58	1	42	36 (9.5)	NA
Sugarman et al., 2021^42^	USA	RCT (I)	6 mo	28 (28)	29	7	0	93	33.7 (9.7)	25.7 (4.1)
Sugarman et al., 2021^42^	USA	RCT (I)	2 yr	28 (28)	79	21	79	0	31.8 (8.6)	24.4 (4.9)
Maldonado et al., 2020^43^	USA	Cohort (III)	2 yr	88 (88)	88	8	0	92	36.9 (13.8)	26.4 (5.2)
Marland et al., 2023^44^	USA	Cohort (IV)	36.9 mo	123 (123)	100	100	0	0	33.2 (11.6)	25 (5)
Flores et al., 2018^45^	USA	Matched cohort (II)	1 yr	39 (39)	59	0	100	0	33.9 (8.1)	25 (3.4)
Flores et al., 2018^45^	USA	Matched cohort (II)	1 yr	39 (39)	59	0	100	0	31.2 (11.1)	23.8 (3.4)
Murata et al., 2017^46^	Japan	Cohort (III)	6 mo, 1‐ and 2 yr	47 (47)	32	0	0	100	28.3 (11.4)	22.1 (2.7)
Murata et al., 2017^46^	Japan	Cohort (III)	6 mo, 1‐ and 2 yr	27 (27)	68	0	0	100	39.7 (6.6)	22.3 (2.7)
Nguyen et al., 2022^47^	USA	Cohort (II)	1 yr	28 (28)	43	100	0	0	31 (6.1)	24.7 (3.1)
You et al., 2020^48^	USA	Cohort (II)	2 yr	168 (168)	54	45	0	55	35.3 (9.6)	25.7 (4.0)
Mygind‐Klavsen et al., 2020^49^	Denmark	Matched cohort (III)	1 yr	189 (189)	53	0	0	100	39.4 (11.8)	NA
Mygind‐Klavsen et al., 2020^49^	Denmark	Matched cohort (III)	1 yr	189 (189)	53	0	0	100	39.3 (11.2)	NA
Kingery et al., 2024^50^	USA	Cohort (III)	5 yr	94 (94)	68	3	15	82	41.9 (14.2)	25.4 (4.3)
Domb et al., 2024^51^	USA	Cohort (III)	10 yr	130 (145)	87	21	55	24	30.3 (12.9)	23.2 (4.3)
Beck et al., 2019^52^	USA	Cohort (III)	2 yr	224 (224)	68	0	6	94	33.7 (12.6)	24.6 (4)
Kierkegaard et al., 2022^53^	Denmark	Cohort (IV)	1 and 5 yr	60 (60)	63	0	8	92	36 (9)	NA
Carton et al., 2021^54^	Ireland	Cohort (IV)	2, 5, and 10 yr	128 (138)	22	0	0	100	32.3 (9.5)	NA
Matsuda et al., 2015^55^	USA	Cohort (III)	3 mo, 1, and 2 yr	15 (18)	33	48	35	17	37.2 (11.3)	28.2 (5.6)
Matsuda et al., 2015^55^	USA	Cohort (III)	3 mo, 1, and 2 yr	127 (127)	67	54	16	30	39.8 (13.5)	26.9 (5)
Özbek et al., 2021^56^	Turkey	Cohort (IV)	3 and 6 mo, 1 and 2 yr	34 (34)	59	74	20	6	32.3 (12.5)	24.9 (4.2)
Mas Martinez et al., 2022^57^	Spain	Cohort (IV)	40.4 mo	52 (52)	100	35	0	65	39.2 (7)	21.6 (2.5)
Mas Martinez et al., 2022^57^	Spain	Cohort (IV)	50.2 mo	104 (104)	0	34	0	66	37.6 (6.1)	25.8 (3.7)
Pansard et al., 202^58^	France	Cohort (IV)	1 yr	197 (197)	40	18	6	76	35 (9)	NA
Flores et al., 2020^59^	USA	Cohort (II)	2 yr	72 (72)	100	35	0	65	34.2 (9.5)	24.9 (4.4)
Flores et al., 2020^59^	USA	Cohort (II)	2 yr	59 (59)	0	34	0	66	35.8 (10.3)	25.5 (3.3)
Fukase et al., 2022^60^	USA	Matched cohort (III)	104.8 mo	157 (157)	69	18	6	76	16.8 (0.37)	21.5 (0.52)
Fukase et al., 2022^60^	USA	Matched cohort (III)	78.4 mo	157 (157)	69	8	1	90	29.8 (1.48)	22.5 (0.42)
Yang et al., 2022^62^	China	Cohort (III)	38.3 mo	482 (482)	62	74	0	26	36.8 (9.9)	22.8 (3.1)
Matsuda et al., 2019^61^	USA	Matched cohort (III)	2 yr	304 (304)	68	50	28	21	34.2 (NA)	24.4 (NA)
Matsuda et al., 2019^61^	USA	Matched cohort (III)	2 yr	84 (84)	61	0	100	0	37.4 (NA)	25.4 (NA)
Bonin et al., 2024^63^	France	RCT (I)	2 yr	49 (49)	10	53	0	47	28.5 (7.5)	23.5 (3)
Bonin et al., 2024^63^	France	RCT (I)	2 yr	51 (51)	14	67	0	33	30.4 (8.4)	23.4 (1.9)
Flores et al., 2018^64^	USA	Cohort (II)	3 and 6 mo, 1 and 2 yr	122 (129)	56	55	20	25	36.2 (11.2)	25.1 (4.1)
Matsuda et al., 2017^65^	USA	Cohort (IV)	3 mo, 1 and 2 yr	69 (69)	46	0	0	100	38.6 (13.3)	NA
Kaplan et al., 2021^66^	USA	Matched cohort (III)	2 yr	16 (16)	75	0	0	100	42.9 (12.2)	26.5 (3.6)
Kaplan et al., 2021^66^	USA	Matched cohort (III)	2 yr	91 (91)	68	0	0	100	38.7 (12.7)	24.8 (4.2)
Filan et al., 2023^67^	Ireland	Cohort (III)	2 yr	131 (262)	46	3	1	96	25.4 (NA)	NA
Yang et al., 2022^69^	China	Cohort (III)	35.7 mo	84 (84)	46	23	1	76	39.3 (9.4)	NA
Brick et al., 2020^68^	New Zealand	Matched cohort (III)	60.4 mo	93 (114)	50	0	11	90	34.5 (12.2)	25.4 (5.1)
Brick et al., 2020^68^	New Zealand	Matched cohort (III)	61.8 mo	108 (114)	45	9	0	91	34.5 (12.2)	24.3 (6.2)
Gicquel et al., 2014^70^	France	Cohort (IV)	10 and 55.2 mo	51 (53)	60	43	19	38	31 (NA)	NA
Bolia et al., 2019^71^	USA	Matched cohort (III)	87.6 mo	42 (42)	42	83	0	0	38 (15)	NA
Bolia et al., 2019^71^	USA	Matched cohort (III)	87.6 mo	84 (84)	42	75	25	0	38 (15)	NA
Nguyen et al., 2020^72^	USA	Cohort (II)	2 yr	166 (166)	55	69	0	31	35.3 (9.6)	25.1 (4.0)
Tahoun et al., 2023^73^	Spain and Egypt	Matched cohort (III)	6 mo, 2 and 5 yr	42 (42)	27	66	9	25	38.2 (9.8)	24.2 (2.6)
Tahoun et al., 2023^73^	Spain and Egypt	Matched cohort (III)	6 mo, 2 and 5 yr	44 (44)	21	57	14	29	35.6 (9.2)	25.6 (2.4)

BMI, body mass index; LOE, level of evidence; mo, month; n, number; RCT, randomized controlled trial; SD, standard deviation; yr, year.

* Due to rounding differences and incomplete reporting in some studies, the sum of CAM, Pincer, and Mixed diagnoses does not equal the total number of patients.

† I, high‐quality RCT; II, lesser‐quality RCT/prospective comparative; III, case‐control/retrospective comparative; IV, case series.

Eligible studies included randomized controlled trials or observational studies with clear evidence of prospective data collection evaluating primary hip arthroscopy for FAIS. Retrospective studies were excluded because retrospective documentation of complications and PROMs is typically incomplete and inconsistently collected, introducing substantial risk of reporting and selection bias.^17^ As randomized controlled trials focusing exclusively on FAIS are few, observational studies were also included to ensure a comprehensive synthesis of available evidence. Studies had to report both complications, including either clinical complications, conversion to total hip arthroplasty (THA), or revision surgery, and PROMs, including baseline and follow‐up values. Studies were excluded if they used only the Harris Hip Score or the modified Harris Hip Score (mHHS), given their lack of content validity and ceiling effects.^15^, ^18^ Studies relying solely on non‐hip‐specific measures, such as the Merle d’Aubigné Score, the University of California, Los Angeles Activity Scale, visual analog scale, Short Form‐36, or the European Quality of Life questionnaire, were also excluded.

Patients had to be diagnosed with either cam morphology (defined as alpha angle ≥ 50°), pincer morphology (defined as lateral center‐edge angle > 39°), or mixed morphology. Furthermore, patients with hip osteoarthritis were excluded (defined as Tönnis grade > 2, Kellgren‐Lawrence score > 2, or lateral joint space width < 2 mm). Studies were excluded if patients had a history of ipsilateral hip surgery, hip fractures, slipped capital femoral epiphysis, Legg‐Calvé‐Perthes disease, spine surgery, borderline hip dysplasia (defined as a lateral center‐edge angle of 20°‐25°), or hip dysplasia (defined as a lateral center‐edge angle < 20°).

### Data Extraction

Two independent reviewers (J.D., F.N.F.) extracted data using a pilot‐tested template. Extracted information included study characteristics, participant demographics, radiographic and clinical variables, surgical details, follow‐up duration, and reported complications and PROMs. Discrepancies were resolved through discussion and consensus. Several publications included more than 1 arthroscopy cohort; matched‐cohort studies reported 2 distinct arthroscopy groups, and some randomized controlled trials included 2 arthroscopy arms. Because the review summarized single‐group outcomes rather than comparative contrasts, each arthroscopy cohort was extracted and treated as an independent study arm.

Complications were extracted as the number of events reported for conversion to THA, revision surgery, nerve paresthesia, and any additional study‐reported complications not captured by these predefined categories. A complication severity classification system was considered during protocol development; however, reporting across included studies was insufficiently detailed to allow consistent application. Therefore, no complication classification framework was used in this review.^11^

Studies were included if they reported hip‐specific PROMs, such as the International Hip Outcome Tool (iHOT), Copenhagen Hip and Groin Outcome Score, Hip Outcome Score, Hip Disability and Osteoarthritis Outcome Score, Non‐Arthritic Hip Score, or Western Ontario and McMaster Universities Arthritis Index. As these instruments assess overlapping constructs, PROM findings were summarized across 5 domains: pain, symptoms, activities of daily living, sports participation, and quality of life, with total or composite scores grouped separately as composite outcomes. All PROMs were either reported on or converted to a 0 to 100 scale, with higher scores indicating better status. Not all studies contributed data to each domain. Domain‐specific summaries, therefore, reflect only studies that report PROMs for that construct. No new combined or algorithm‐derived PROM score was created, and no pooled meta‐analysis was conducted across instruments; instead, all summaries represent descriptive, construct‐level patterns of change.

### Risk‐of‐Bias Assessment

Risk of bias was independently evaluated by 3 reviewers (F.N.F., I.M., L.U.T.) using the Risk of Bias in Non‐randomized Studies of Interventions (ROBINS‐I) tool, Version 2.^19^ A risk‐of‐bias assessment was conducted for both complications and PROMs using a pilot‐tested form. Discrepancies among reviewers were resolved through consensus discussion, and when agreement could not be reached, a third reviewer provided adjudication.

The ROBINS‐I tool evaluated 7 domains: confounding (D1), participant selection (D2), classification of interventions (D3), deviations from intended interventions (D4), missing data (D5), measurement of outcomes (D6), and selection of reported results (D7). Each domain was judged to have low, moderate, serious, or critical risk of bias using the ROBINS‐I algorithm. An overall risk of bias was assessed in accordance with the ROBINS‐I guidelines. Critical confounding factors were prespecified during the planning stage, including age and hip morphology, given their potential to substantially bias the intervention‐outcome effect estimate.^8^ Because the review summarized single‐group outcomes rather than randomized treatment contrasts, arthroscopy arms originating from randomized controlled trials were treated as prospective cohorts and evaluated using the same ROBINS‐I framework applied to non‐randomized studies. An online implementation of ROBINS‐I was used to visualize the risk‐of‐bias assessment.^20^

### Grading of Recommendations, Assessment, Development, and Evaluation

The Grading of Recommendations, Assessment, Development, and Evaluation (GRADE) framework was used to assess the certainty of evidence for each subjective synthesis of outcomes (i.e., complications and PROMs) following hip arthroscopy for FAIS.^21^, ^22^ Certainty ratings range from very low to high, reflecting confidence in the effect estimates. In single‐arm prospective cohort studies lacking a comparison group, evidence is of low certainty due to limitations in causal inference. Certainty may be upgraded for (1) large effect sizes (e.g., PROM improvements exceeding the minimal clinically important difference [MCID]), (2) a dose‐response gradient, or (3) residual confounding likely to attenuate the observed effect. Certainty may be downgraded for risk of bias, imprecision (e.g., range for PROM changes spanning the MCID or for complication proportions including clinically significant thresholds), (3) inconsistency (e.g., *I* ^2^ ≥ 25% in meta‐analyses), (4) indirectness, or (5) publication bias (e.g., predominance of small studies with positive outcomes illustrated with funnel plots).^23^, ^24^, ^25^, ^26^, ^27^ To support the interpretation of PROM changes, established MCIDs for composite scores were used as reference values. Published MCIDs for the iHOT‐33 (6‐11 points), Non‐Arthritic Hip Score (8.4‐9.4 points), and Western Ontario and McMaster Universities Arthritis Index total score (7.0‐9.0 points) were sufficiently similar to guide interpretation of overall or composite PROM change.^18^, ^28^, ^29^ For individual PROM domains, where reported MCIDs showed substantially greater variability across instruments, a 10‐point threshold was applied as a GRADE‐based interpretive benchmark for assessing the magnitude and precision of change. This threshold was used only for interpretation and was not derived using distribution‐based methods.^29^

### Statistical Analysis

All analyses were conducted in STATA (Version 18.1, StataCorp LLC, College Station, TX, USA).

Standard deviations were estimated when not reported, using established methods.^30^, ^31^ For complications, unweighted proportions and their ranges were calculated, representing the simple mean and spread of study‐specific proportions. This approach provides a straightforward, descriptive estimate across studies, treating each study equally regardless of sample size. For PROMs, mean changes were synthesized as the range of mean changes from baseline to follow‐up. Heterogeneity was assessed and reported using *I* ^2^ (the proportion of total variability attributable to between‐study heterogeneity).^31^ The primary analysis of complication proportions and mean changes in PROMs was conducted irrespective of follow‐up duration.

Subgroup analyses were conducted to describe complications and PROMs of hip arthroscopy for FAIS by follow‐up duration and capsular management. For PROMs, mean changes were analyzed at 3 months, 6 months, 1 year, 2 years, 5 years, and 10 years to describe temporal trends in outcomes. For complications, unweighted proportions were reported by follow‐up period (<2 years vs >2 years) to assess time‐dependent risks. In addition, outcomes were analyzed separately for studies in which patients underwent capsular repair (e.g., suturing or plication) or no repair, providing further insight into the influence of capsular management.

## RESULTS

**FIGURE 1:**
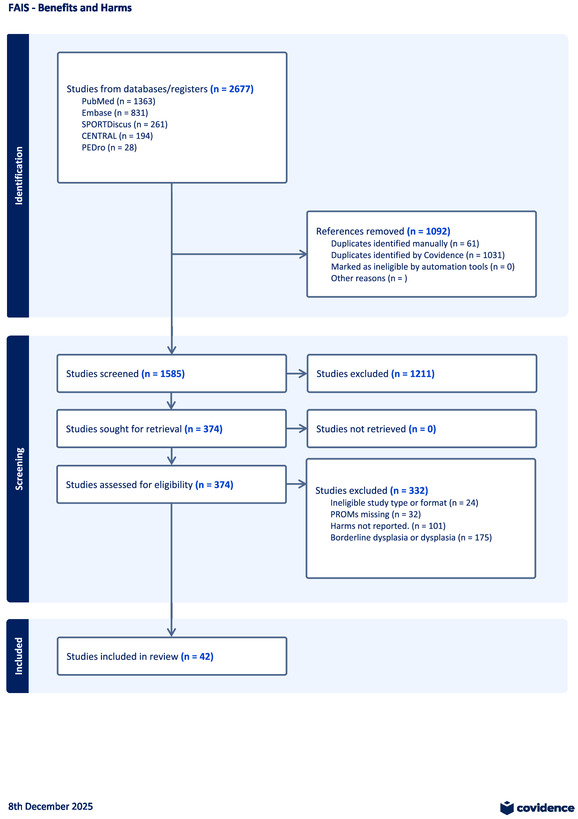
Literature search using the Preferred Reporting Items for Systematic Reviews and Meta‐Analyses guidelines. (PROMs, patient‐reported outcome measures.)

The systematic literature search identified 2677 records, of which 1092 were duplicates. The remaining 1585 records were screened by title and abstract, and 1211 were excluded. A full‐text assessment of 374 articles resulted in the exclusion of 332 studies. Ultimately, 42 studies, comprising 58 study arms, 5646 unique patients, and 5885 hips were included in the systematic review and subjective synthesis (Figure 1).^32^, ^33^, ^34^, ^35^, ^36^, ^37^, ^38^, ^39^, ^40^, ^41^, ^42^, ^43^, ^44^, ^45^, ^46^, ^47^, ^48^, ^49^, ^50^, ^51^, ^52^, ^53^, ^54^, ^55^, ^56^, ^57^, ^58^, ^59^, ^60^, ^61^, ^62^, ^63^, ^64^, ^65^, ^66^, ^67^, ^68^, ^69^, ^70^, ^71^, ^72^, ^73^

Sample sizes ranged from 15 to 482 patients and 16 to 482 hips, totaling 5646 patients and 5885 hips. Of the 5646 patients included, 239 (4.2%) underwent bilateral hip arthroscopy. The mean age was 34.5 years (standard deviation [SD] 11.7; range 16.8‐62.3), with 54% of participants female, and a mean body mass index of 24.3 kg/m^2^ (SD 3.2). Hip morphology was reported as 32% cam, 11% pincer, and 55% mixed, with all hips classified as Tönnis grade 0 to 1 (Table 1). Supplementary treatments, most commonly femoroplasty, labral repair, and acetabuloplasty, were frequently performed in conjunction with hip arthroscopy (Table 2).

**TABLE 2:** Supplementary Treatments

Supplementary Treatment	Study Arms, n	Hips, n	%
Femoroplasty	52	5147	88.4
Acetabuloplasty	50	3890	68.4
Labral repair	53	4544	79.9
Labral debridement	25	361	6.4
Labral reconstruction	20	184	3.2
Capsular repair, suturing, or plication	28	2350	41.3
Synovectomy	4	513	9.0
Chondroplasty	12	476	8.4
Ligamentum teres treatment	11	462	8.1
Microfracture	23	220	3.9
Iliopsoas fractional lengthening	6	160	2.8
Subspine decompression	5	65	1.1
Psoas tenotomy	1	28	0.5
Trochanteric bursectomy	2	14	0.2
Removal of foreign bodies	1	2	<0.1

%, percentage of included hips that received the supplementary treatment; n, number.

**FIGURE 2:**
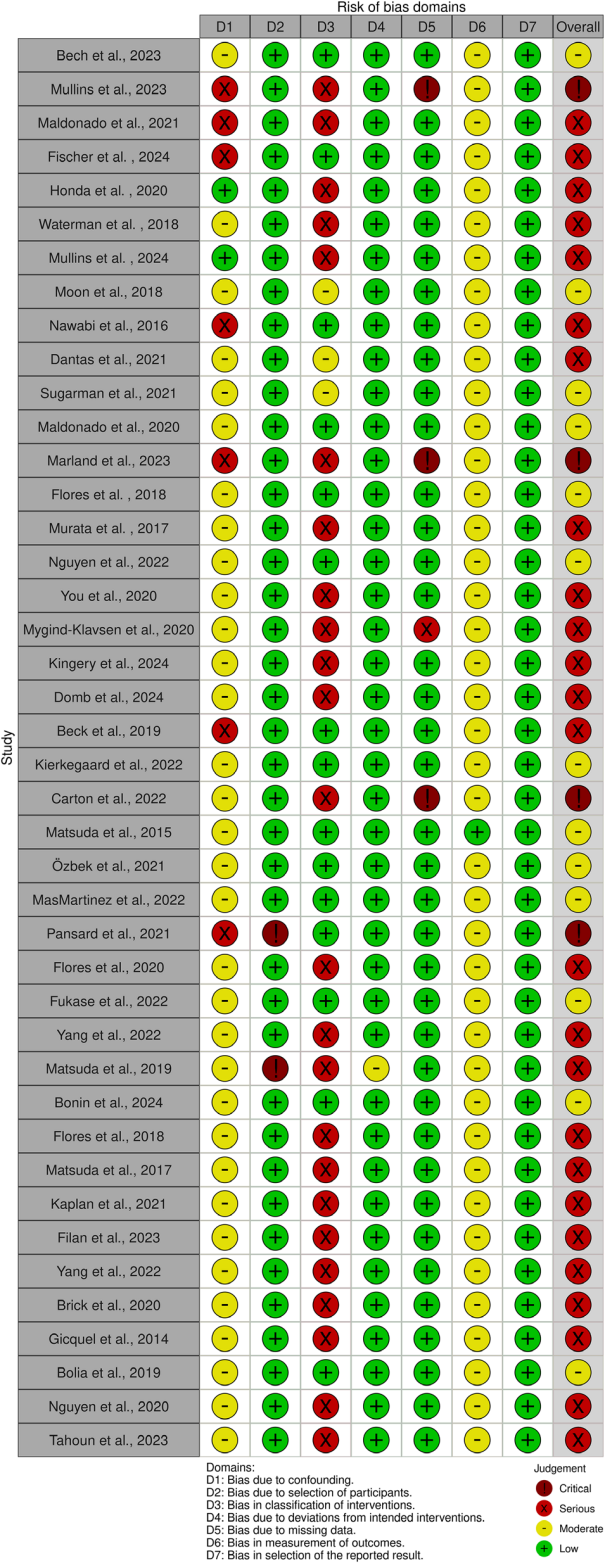
ROB assessment of complications in each study using the Cochrane ROBINS‐I tool. ROBINS‐I: D1 to D7: Domain 1 to Domain 7. (ROB, risk of bias; ROBINS‐I, Risk of Bias in Non‐randomized Studies of Interventions.)

**FIGURE 3:**
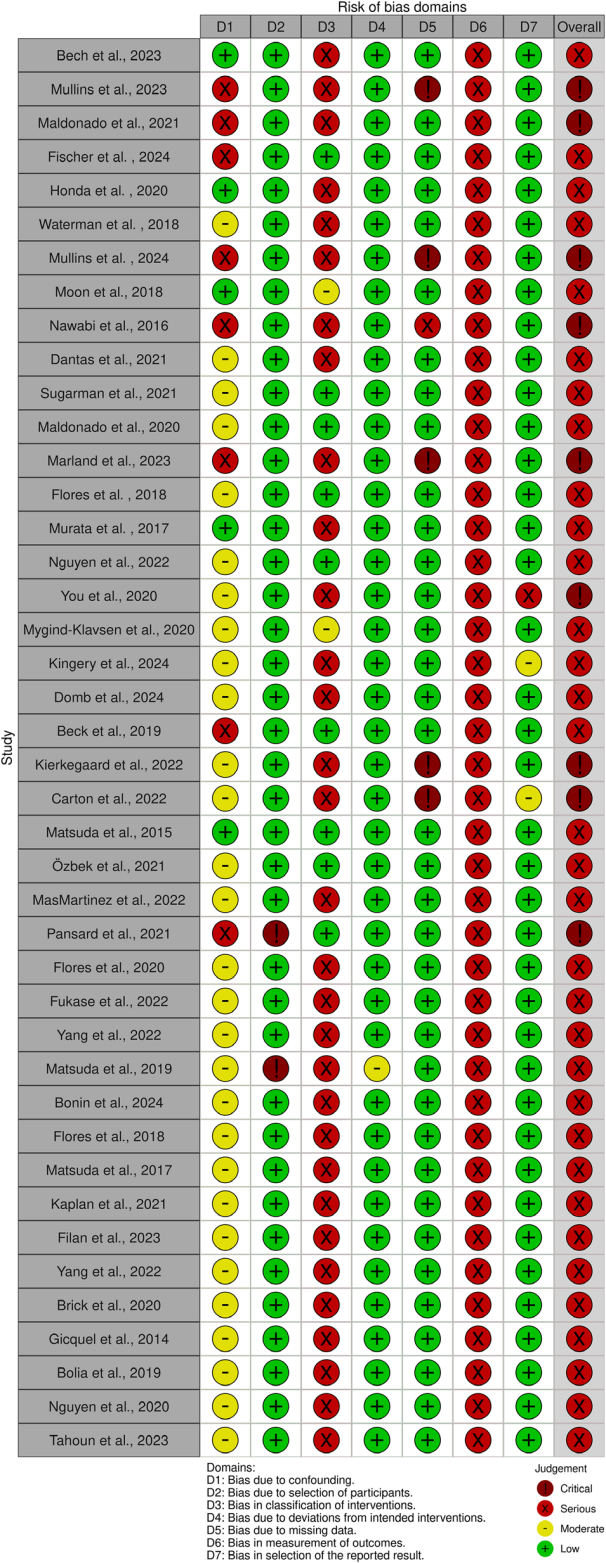
ROB assessment of patient‐reported outcome measures in each study using the Cochrane ROBINS‐I tool. ROBINS‐I: D1 to D7: Domain 1 to Domain 7. (ROB, risk of bias; ROBINS‐I, Risk of Bias in Non‐randomized Studies of Interventions.)

The overall risk of bias regarding complications was rated as moderate in 19 (33%), serious in 36 (62%), and critical in 3 (5%) study arms (Figure 2). For PROMs, the risk of bias was rated as serious in 49 (84%) and critical in 9 (16%) study arms (Figure 3).

### Complications

Proportions of complications following hip arthroscopy are presented in Table 3 and Figures S1‐S4. The unweighted proportions were 2.1% (range, 0%‐16.7%) for conversion to THA and 5.2% (range, 0%‐22.3%) for revision surgery. Overall, nerve paresthesia occurred in 3.8% of hips (range 0.8%‐12.5%). Pudendal neuropraxia occurred in 2.6% of hips (range 0.8%‐7.5%) and lateral femoral cutaneous nerve neuropraxia in 1.9% (range 1.2%‐5.1%), with sciatic and unspecified neuropraxia reported only rarely.

**TABLE 3:** Summary of Systematic Review on the Complications of Hip Arthroscopy Among Patients With FAIS, Including Unweighted Proportions and *I* ^2^ to Measure Heterogeneity

Complication	Study Arms, n	Events, n	Hips, n*	Unweighted Proportion (Range %)	*I* ^2^ †
**Total hip arthroplasty**	58	110	5267	2.1% (0%‐16.7%)	74%
**Revision surgery**	58	273	5267	5.2% (0%‐22.3%)	82%
**Temporary nerve paresthesia**	10	42	1106	3.8% (0.8%‐12.5%)	59%
Pudendal neuropraxia	7	22	854	2.6% (0.8%‐7.5%)	
Lateral femoral cutaneous neuropraxia	5	11	594	1.9% (1.2%‐5.1%)	
Sciatic neuropraxia	1	1	90	1.1% (1.1%‐1.1%)	
Unspecified neuropraxia	1	8	64	12.5% (12.5%‐12.5%)	
**Other complications**	5	7	488	1.4% (0.6%‐2.9%)	
Tendinopathy	2	2	100	2.0% (2.0%‐2.0%)	
Asymptomatic heterotopic ossification	2	3	289	1.0% (0.6%‐1.6%)	
Algodystrophy	1	2	49	4.1% (4.1%‐4.1%)	
Deep gluteal syndrome	2	2	228	0.9% (0.9%‐0.9%)	
Anterior hip dislocation	1	1	160	0.6% (0.6%‐0.6%)	
**Total**	58	484	5267	9.2% (0.6%‐12.5%)	

FAIS; femoroacetabular impingement syndrome; n, number.

* Total hips reflect the full review sample for complications: fewer hips for some outcomes due to incomplete reporting or dropouts in the included studies.

† Nerve paresthesia includes pudendal, lateral femoral cutaneous, sciatic, and unspecified neuropraxia. Unweighted proportions and ranges (%).

The mean time from primary hip arthroscopy to conversion to THA, when including all available studies (with imputed last follow‐up timepoints if no mean was reported), was 45.7 months (SD 31.6) based on 27 studies and 110 conversion events. When restricted to studies that explicitly reported mean values, the mean time to THA conversion was 52.6 months (SD 32.3) across 5 studies, with 69 events. For revision arthroscopy, the mean time from primary hip arthroscopy to revision was 31.1 months (SD 21.6), derived from 38 studies and 273 revision events. Considering only studies that explicitly reported mean values, the weighted mean time to revision was 28.1 months (SD 26.2) across 9 studies and 108 events.

Subgroup analyses of the effect of follow‐up duration on the proportions converting to THA and undergoing revision surgery are shown in Figures S5‐S8. The unweighted conversion rate to THA was 1.4% (range 0%‐10.1%) within 2 years and 5.4% (range 0%‐16.7%) after 2 years. The unweighted proportion of revision surgery was 2.3% (range, 0%‐14.8%) within 2 years and 9.3% (range, 0%‐22.3%) after 2 years. Subgroup analyses of the effect of capsular repair on unweighted complication proportions are shown in Figures S9‐S12. The unweighted conversion rate to THA was 3% (0%‐16.7%) without capsular repair and 1.8% (0%‐9%) with repair. The unweighted proportion of revision surgery was 5.1% (0%‐22.3%) without capsular repair, and 6.8% (0%‐15.9%) with capsular repair. All subjective syntheses of complications showed high heterogeneity (*I* ^2^ > 50%).

Only 23 study arms reported reasons for revision surgery, with the most common being adhesions (4.6%), labral pathology (3.8%), and residual impingement (1.7%), as shown in Table S6.

### Patient‐Reported Outcomes

The range of mean changes from baseline to follow‐up across hip‐related outcomes is presented in Figures S13‐S18. The mean change ranged from 9.4 to 35.0 points for pain, 14.3 to 25.0 points for symptoms, 10.9 to 43.0 points for activities of daily living, 15.0 to 45.5 points for sports participation, 15.0 to 43.2 points for quality of life, and 10.3 to 47.6 points for composite scores. All subjective syntheses of mean changes from baseline to follow‐up across constructs showed substantial heterogeneity (*I* ^2^ > 50%).

Mean changes in PROMs were described separately by follow‐up duration (Figures S19‐S24) and by capsular repair status (Figures S25‐S30).

### Grading of Recommendations, Assessment, Development, and Evaluation

The overall quality of the evidence, as assessed using GRADE, including the reasons for downgrading, is summarized in Table 4. Funnel plots for each outcome are depicted in Figures S31‐S32. The certainty of evidence was rated very low for all outcomes, including complications (conversion to THA and revision surgery) and PROMs (pain, activities of daily living, sports participation, quality of life, symptoms, and composite scores).

**TABLE 4:** GRADE Summary of Findings Table for Conversion to Hip Arthroplasty, Revision Surgery, and Patient‐Reported Outcome Measures

Certainty Assessment							No. of Patients		Effect		Certainty	Importance
No. of Study Arms	Study Design	Risk of Bias	Inconsistency	Indirectness	Imprecision	Other Considerations	Hip Arthroscopy	Not Applicable (Pre‐Post for PROMs; Single‐Arm Incidence for Complications)	Relative (95% CI)	Absolute (95% CI)		
Conversion to total hip arthroplasty												
58*	Non‐randomized studies	Very serious†	Serious‡	Not serious$	Serious||	None¶	110/5267 (2.1%)		Not estimable	Not estimated	⊕◯◯◯Very low†, ‡, $, ||, ¶	CRITICAL
Revision surgery												
58*	Non‐randomized studies	Very serious†	Very serious#	Not serious$	Serious||	None¶	273/5267 (5.2%)		Not estimable	Not estimated	⊕◯◯◯ Very low†, $, ||, ¶, #	CRITICAL
Pain (assessed with HAGOS and HOOS; scale from 0 to 100)												
19*	Non‐randomized studies	Extremely serious∗∗	Very serious††	Not serious$	Serious‡‡	Strong association¶, $$	1786		‐	Range (9.4 higher to 35 higher)||||	⊕◯◯◯ Very low$, ¶, ∗∗, ††, ‡‡, $$	IMPORTANT
Activities of daily living (assessed with HAGOS, HOOS, and HOS; scale from 0 to 100)												
34*	Non‐randomized studies	Extremely serious∗∗	Very serious††	Not serious$	Not serious¶¶	Strong association¶, $$	2587		‐	Range (10.9 higher to 43 higher)||||	⊕◯◯◯ Very low$, ¶, ∗∗, ††, $$, ¶¶	IMPORTANT
Sports participation (assessed with HAGOS, HOOS, and HOS; scale from 0 to 100)												
36*	Non‐randomized studies	Extremely serious∗∗	Very serious††	Not serious$	Not serious¶¶	Strong association¶, $$	2754		‐	Range (15 higher to 45.5 higher)||||	⊕◯◯◯ Very low$, ¶, ∗∗, ††, $$, ¶¶	IMPORTANT
Quality of life (assessed with HAGOS and HOOS; scale from 0 to 100)												
19*	Non‐randomized studies	Extremely serious∗∗	Very serious††	Not serious$	Not serious¶¶	Strong association¶, $$	1786		‐	Range (15 higher to 43.2 higher)||||	⊕◯◯◯ Very low$, ¶, ∗∗, ††, $$, ¶¶	IMPORTANT
Symptoms (assessed with HAGOS and HOOS; scale from 0 to 100)												
19*	Non‐randomized studies	Extremely serious∗∗	Serious##	Not serious$	Not serious¶¶	Strong association¶, $$	1786		‐	Range (14.3 higher to 25 higher)||||	⊕◯◯◯ Very low$, ¶, ∗∗, $$, ¶¶, ##	IMPORTANT
Composite score (assessed with iHOT‐12, iHOT‐33, NAHS, and WOMAC; scale from 0 to 100)												
42*	Non‐randomized studies	Extremely serious∗∗	Very serious††	Not serious	Not serious¶¶	Strong association¶, $$	3582		‐	Range (10.3 higher to 47.6 higher)||||	⊕◯◯◯ Very low¶, ∗∗, ††, $$, ¶¶	IMPORTANT

CI, confidence interval; FAIS, femoroacetabular impingement syndrome; GRADE, Grading of Recommendations, Assessment, Development, and Evaluation; HAGOS, Hip and Groin Outcome Score; HOS, Hip Outcome Score; HOOS, Hip Disability and Osteoarthritis Outcome Score; iHOT, International Hip Outcome Tool; MCID, minimal clinically important difference; NAHS, Non‐Arthritic Hip Score; PROMs, patient‐reported outcome measures; ROBINS‐I, Risk of Bias in Non‐randomized Studies of Interventions; THA, total hip arthroplasty; WOMAC, Western Ontario and McMaster Universities Arthritis Index.

* Refer to the number of study arms, as some trials had more than 1 study arm (matched cohorts or randomized controlled trials) with FAIS patients undergoing hip arthroscopy.

† The overall risk of bias was moderate for 19 (33%), serious for 36 (62%), and critical for 3 (5%) study arms regarding complications (Figure 2).

‡ Heterogeneity: *I* ^2^ = 50.4%.

$ Inclusion and exclusion criteria have ensured a homogeneous population of FAIS patients undergoing hip arthroscopy.

|| We did not identify studies that explicitly define clinically relevant thresholds for absolute proportions of conversion to THA or revision surgery in patients undergoing hip arthroscopy for FAIS. The ranges of proportions for THA were 0% to 16.7% and 0% to 22.3% for revision surgery. Accordingly, we downgraded due to imprecision.

¶ Publication bias, not detected. See funnel plots in the supplementary materials. We tested the null hypothesis that there is no small‐study effect (i.e., that the funnel plot is symmetrical). There was no indication of publication bias or small‐study effects in the meta‐analysis of complications or patient‐reported outcomes (i.e., *P* values > .05).

# Heterogeneity: *I* ^2^ = 79.0%.

∗∗ ROBINS‐I Version 2 tool: the overall risk of bias was serious for 49 (84%) study arms and critical for 9 (16%) study arms regarding PROMs (Figure 3).

†† Heterogeneity: *I* ^2^ > 50% (e.g., pain = 82.1%, activities of daily living = 91.3%, sports participation = 93.8%, quality of life = 92.9%, composite score = 96.6%).

‡‡ Downgraded based on imprecision: range of mean effects down to 9.4 points (e.g., lower than HAGOS subscales MCID of 10 points).

$$ Large effect as the lower bound of the range intervals exceeds the established MCID threshold.

|||| The reported absolute effects represent the study‐specific mean changes from baseline to follow‐up across included studies. The range is the difference between the lowest and highest effect reported. Follow‐up durations varied across studies; mean effects are reported irrespective of follow‐up time.

¶¶ Not downgraded based on imprecision, as the lower bound of the range exceeds the established MCID thresholds (e.g., iHOT‐33 ≥ 6 to 11, HAGOS subscales ≥ 10). This indicates high certainty that effects are clinically meaningful.

## Heterogeneity: *I* ^2^ < 50% (e.g., 49.6%).

## DISCUSSION

Across the included prospective studies, hip arthroscopy for FAIS was associated with low reported proportions of major complications and consistent improvements across all PROM domains.

### Complications

This systematic review and subjective synthesis found that conversion to THA and revision arthroscopy occurred in a small proportion of patients following hip arthroscopy for FAIS, with unweighted proportions slightly lower than those reported in previous reviews. The unweighted proportions were 2.1% (range 0%‐16.7%) for THA and 5.2% (range 0%‐22.3%) for revision arthroscopy, whereas earlier reviews reported 2.9% to 6.3% for THA and 6.3% for revision surgery.^9^, ^74^

A longer follow‐up duration was associated with higher proportions of both THA and revision arthroscopy, supporting the relevance of extended follow‐up for capturing delayed secondary surgery. For instance, conversion to THA increased from 1.4% (range 0%‐10.1%) within 2 years to 5.6% (range 0%‐16.7%) beyond 2 years. Similarly, revision arthroscopy rose from 2.3% (range 0%‐14.8%) to 9.3% (0%‐22.3%) over the same time frame. These findings emphasize that secondary surgery may not be fully apparent in short‐term studies and that long‐term follow‐up is essential for accurate assessment of surgical durability.

Surgical techniques may also influence the need for secondary surgery. Labral repair was performed in nearly 80% of patients across the included studies, which may have contributed to lower revision rates. Previous studies have linked labral repair to improved outcomes and reduced risk of revision surgery.^75^ For capsular repair, the unweighted proportions were 1.8% (range 0%‐9%) for conversion to THA and 6.8% (range 0%‐15.9%) for revision arthroscopy, compared with 3% (range 0%‐16.7%) and 5.1% (range 0%‐22.3%) without repair, respectively. Although previous evidence suggests that capsular repair may enhance joint stability and reduce the risk of secondary surgery, the present findings are conflicting.^76^, ^77^ High‐quality studies with longer follow‐up are needed to clarify the role of capsular management.

The most frequently reported causes of revision surgery were adhesions (4.6%) and persistent labral pathology (3.8%). These numbers were based solely on studies that explicitly reported such complications and may therefore be overestimated due to selective reporting. Adhesions and residual labral damage may reflect limitations in surgical access or postoperative healing, but interpretation is constrained by inconsistent documentation and limited detail on rehabilitation protocols.^78^, ^79^

Nerve paresthesia was the most common clinical complication, with an unweighted proportion of 3.8% (range 0.8%‐12.5%). When disaggregated, pudendal neuropraxia occurred in 2.6% of hips (range 0.8%‐7.5%) and lateral femoral cutaneous nerve neuropraxia in 1.9% (range 1.2%‐5.1%), with sciatic and unspecified neuropraxia reported only rarely. These events were generally temporary, although substantial variability in reporting was observed. A sensitivity analysis assuming that studies not reporting nerve paresthesia had zero events yielded an unweighted proportion of 0.8% (range 0%‐12.5%), suggesting potential underreporting. This concern is supported by registry‐based studies such as Gilinov et al.,^12^ who reported an incidence of 0.22%, and by Wininger et al.,^80^ who found that prospective studies reported pudendal neuropraxia more frequently than retrospective studies. Together, these patterns indicate that pudendal and lateral femoral cutaneous nerve neuropraxia may be more common than suggested by retrospective cohorts and that the true incidence of nerve‐related complications is likely underestimated in the current literature.

Our findings suggest that hip arthroscopy for FAIS is associated with low proportions of secondary surgery and clinical complications, including conversion to THA, revision arthroscopy, and nerve paresthesia. These findings likely reflect advances in surgical technique, improved patient selection, and growing surgeon experience. Although our review did not evaluate temporal trends, contemporary registry data suggest minor improvements in 1‐year PROMs over the past decade, suggesting modest gains in postoperative function.^81^ Nevertheless, the potential for underreporting remains a concern, particularly for clinical complications. Greater use of prospective data collection, longer‐term follow‐up, and consistent definitions for reporting complications are necessary to characterize the safety profile of hip arthroscopy for FAIS fully.

### Patient‐Reported Outcomes

PROMs improved across all domains, with mean changes from baseline to follow‐up ranging from 9.4 to 35.0 points for pain, 14.3 to 25.0 for symptoms, 10.9 to 43.0 for activities of daily living, 15.0 to 45.5 for sports participation, 15.0 to 43.2 for quality of life, and 10.3 to 47.6 for composite scores, with most exceeding established thresholds for clinically meaningful improvement.

Our findings are consistent with those reported in a 2019 systematic review and meta‐analysis by Minkara et al.,^8^ who also observed considerable and clinically meaningful improvements in PROMs following hip arthroscopy for FAIS. However, our review differs in several key respects. Including studies published after 2018, it reflects more recent surgical practices and outcome reporting. Minkara et al. primarily included studies using the mHHS; however, our review focused on studies employing validated hip‐specific PROMs, such as iHOT, Hip and Groin Outcome Score, and Hip Disability and Osteoarthritis Outcome Score. Although the mHHS remains widely used, it has limitations, including ceiling effects and limited content validity in young, active populations.^82^ For this reason, studies relying solely on the mHHS were excluded. This approach aligns with the 2016 Warwick Agreement, which recommends the use of iHOT, Hip and Groin Outcome Score, or Hip Disability and Osteoarthritis Outcome Score to evaluate outcomes following hip arthroscopy for FAIS.^1^ The extended time frame of our review also increased the number of available studies for each PROM. Despite these methodological strengths, the overall certainty of the evidence for PROMs was rated as very low, primarily due to heterogeneity and loss to follow‐up in the risk‐of‐bias assessment. This may be related to both the intervention and the outcomes, as patients with complications (i.e., conversion to THA, revision surgery, or clinical complications) or limited improvements in PROMs could be less likely to complete follow‐up assessments, introducing a risk of attrition bias and potentially inflating estimates of treatment effects on PROMs.

### Limitations

This systematic review has several methodological limitations that warrant consideration. First, we included only studies with prospectively collected data, a clearly defined diagnosis of FAIS, and reporting of both complications and PROMs. This ensured that complications and PROM changes were derived from the same cohort and avoided indirect comparisons between different study populations. Because prospective data collection was most transparently reported in studies that used serial PROMs, this criterion also reduced the risk of including studies with incomplete or inconsistently collected complication data. However, it excluded studies that reported only one of these domains, thereby reducing the breadth of available evidence. Second, the overall risk of bias across included studies was frequently rated as serious. For PROM outcomes in particular, the lack of blinding of patients and assessors contributed to uniformly serious or critical ratings. Although a proportion of complication‐related outcomes were rated as moderate, serious concerns remained common. These patterns reduce confidence in the observed estimates and are consistent with the very low GRADE certainty assigned to all outcomes. Third, substantial between‐study heterogeneity in surgical techniques, rehabilitation protocols, PROM selection, and follow‐up intervals precluded robust quantitative synthesis. Finally, although subgroup analyses were conducted for follow‐up duration and capsular management, sex‐specific effects could not be evaluated because only 2 studies reported sex‐disaggregated outcomes, preventing meaningful comparisons.

## CONCLUSIONS

Hip arthroscopy for FAIS is associated with low average rates of THA conversion, revision surgery, and nerve paresthesia and with clinically meaningful improvements across PROM domains. However, the certainty of evidence is very low, and results should be interpreted with caution.

## DECLARATION OF GENERATIVE AI AND AI‐ASSISTED TECHNOLOGIES IN THE WRITING PROCESS

During the preparation of this work, the authors used xAI's Grok (Version 3) and OpenAI's ChatGPT (Version GPT‐4o) to draft the manuscript and, by 1 author, to assist with data extraction. After using these tools/services, the authors reviewed and edited the content as needed, verified all data extraction, and take full responsibility for the publication's content.

## SUPPORTING INFORMATION

Additional supporting information can be found online in the Supporting Information section.

## DISCLOSURES

The author (I.M.) declares the following financial interests/personal relationships which may be considered as potential competing interests: I.M. reports financial support provided by Aarhus University Hospital; reports a relationship with Danish Hip Arthroplasty Registry that includes board membership. The other authors (F.N.F., J.D., L.U.T., B.M‐K., C.G., B.Z.) declare that they have no known competing financial interests or personal relationships that could have appeared to influence the work reported in this article.

## Supporting information

Supplementary Material
