# Supplementary material for: Improvements in Patient‐Reported Outcomes and Few Reported Major Complications Following Hip Arthroscopy in Patients With Femoroacetabular Impingement Syndrome: A Systematic Review
Source: Arthrosc Sports Med Rehabil. 2026 Jun 8;8(4):e70036. doi: 10.1002/ars2.70036 (PMC13399726; doi:10.1002/ars2.70036)

Table of contents

Systematic Search Strategies for All Databases 4

Supplementary Table 1. MEDLINE 4

Supplementary Table 2. Embase 5

Supplementary Table 3. PEDro 5

Supplementary Table 4. SPORTDiscus 6

Supplementary Table 5. Cochrane (CENTRAL) 6

Supplementary treatment 7

Supplementary Table 6. Summary of the harms of hip arthroscopy among patients with FAIS, including unweighted proportions, in studies reporting revision surgery, and the reason for revision surgery. 7

Harms, irrespective of follow-up duration. 8

Supplementary Figure 1. Proportion of conversion to total hip arthroplasty following primary hip arthroscopy in patients with femoroacetabular impingement. 8

Supplementary Figure 2. Proportion of revision surgery for primary arthroscopy in patients with femoroacetabular impingement. 9

Supplementary Figure 3. Proportion of nerve paresthesia for primary hip arthroscopy in patients with FAIS, in studies reporting at least one such event. 10

Supplementary Figure 4. Proportion of nerve paresthesia for primary hip arthroscopy in patients with FAIS. 11

Harms, sub-analysis of follow-up duration 12

Supplementary Figure 5. Proportion of conversion to total hip arthroplasty for primary hip arthroscopy in patients with femoroacetabular impingement. Follow-up duration up to 2 years. 12

Supplementary Figure 6. Proportion of conversion to total hip arthroplasty for primary hip arthroscopy in patients with femoroacetabular impingement. Follow-up duration after 2 years. . 13

Supplementary Figure 7. Proportion of revision surgery for primary hip arthroscopy in patients with femoroacetabular impingement. Follow-up duration up to 2 years. 14

Supplementary Figure 8. Proportion of revision surgery for primary hip arthroscopy in patients with femoroacetabular impingement. Follow-up duration after 2 years. 15

Harms, sub-analysis of capsular closure versus no capsular closure. 16

Supplementary Figure 9. Proportion of conversion to total hip arthroplasty for primary hip arthroscopy in patients with femoroacetabular impingement. Capsular repair. 16

Supplementary Figure 10. Proportion of conversion to total hip arthroplasty for primary hip arthroscopy in patients with femoroacetabular impingement. No capsular repair. 17

Supplementary Figure 11. Proportion of revision surgery for primary hip arthroscopy in patients with femoroacetabular impingement. Capsular repair. 18

Supplementary Figure 12. Proportion of revision surgery for primary hip arthroscopy in patients with femoroacetabular impingement. No capsular repair. 19

Benefits across hip-related outcomes, irrespective of follow-up duration. 20

Supplementary Figure 13. Change of Pain subscales from baseline to each follow-up, after primary hip arthroscopy for patients with femoroacetabular impingement. 20

Supplementary Figure 14. Change in the Activities of Daily Living subscales from baseline to each follow-up, after primary hip arthroscopy for patients with femoroacetabular impingement. 21

Supplementary Figure 15. Change in the Sports Participation subscales from baseline to each follow-up, after primary hip arthroscopy for patients with femoroacetabular impingement. 22

Supplementary Figure 16. Change in the Quality-of-Life subscales from baseline to each follow-up, after primary hip arthroscopy for patients with femoroacetabular impingement. 23

Supplementary Figure 17. Change in the Symptoms subscales from baseline to each follow-up, after primary hip arthroscopy for patients with femoroacetabular impingement. 24

Supplementary Figure 18. Change in the composite Total scores from baseline to each follow-up, after primary hip arthroscopy for patients with femoroacetabular impingement. 25

Benefits across hip-related outcomes, including a sub-analysis of follow-up durations of 3- and 6 months, 1-, 2-, 5-, and 10-year follow-ups. 26

Supplementary Figure 19. Change in the Pain subscales from baseline to each follow-up for primary hip arthroscopy in patients with femoroacetabular impingement. 26

Supplementary Figure 20. Change in the Activities of Daily Living subscales from baseline to each follow-up, after primary hip arthroscopy in patients with femoroacetabular impingement. 27

Supplementary Figure 21. Change in the Sports Participation subscales from baseline to each follow-up, after primary hip arthroscopy in patients with femoroacetabular impingement. 28

Supplementary Figure 22. Change in the Quality-of-Life subscales from baseline to each follow-up, after primary hip arthroscopy in patients with femoroacetabular impingement. 29

Supplementary Figure 23. Change in the Symptoms subscale from baseline to each follow-up, after primary hip arthroscopy in patients with femoroacetabular impingement. 30

Supplementary Figure 24. Change in the composite Total score from baseline to each follow-up, after primary hip arthroscopy in patients with femoroacetabular impingement. 31

Benefits across hip-related outcomes, sub-analysis of Capsular Repair or no Capsular Repair. 32

Supplementary Figure 25. Change in Pain subscales from baseline to each follow-up, after primary hip arthroscopy in patients with femoroacetabular impingement. Capsular repair or no capsular repair. 32

Supplementary Figure 26. Mean change in the Activities of Daily Living subscales from baseline to each follow-up, after primary hip arthroscopy in patients with femoroacetabular impingement. Capsular repair or no capsular repair. 33

Supplementary Figure 27. Mean change in the Sports Participation subscales from baseline to each follow-up, after primary hip arthroscopy in patients with femoroacetabular impingement. Capsular repair or no capsular repair. 34

Supplementary Figure 28. Mean change in the Quality-of-Life subscales from baseline to each follow-up, after primary hip arthroscopy in patients with femoroacetabular impingement. Comparing Capsular repair or no capsular repair. 35

Supplementary Figure 29. Mean change in the Symptoms subscales from baseline to each follow-up, after primary hip arthroscopy in patients with femoroacetabular impingement. Comparing Capsular repair or no capsular repair. 36

Supplementary Figure 30. Mean change in the composite Total scores from baseline to each follow-up, after primary hip arthroscopy in patients with femoroacetabular impingement. Comparing Capsular repair or no capsular repair. 37

Funnel plots. 38

Supplementary Figure 31. Funnel plot of studies reporting incidence of conversion to total hip arthroplasty and revision arthroscopy following hip arthroscopy........ 38

Supplementary Figure 32. Funnel plot of studies reporting patient-reported outcome measures. 39

Systematic Search Strategies for All Databases

Supplementary Table 1. MEDLINE

**Line**	**Concept**	**Search Terms**
1	Femoroacetabular Impingement (MeSH)	"Femoracetabular Impingement"/
2	Femoroacetabular Impingement (Keywords)	(femoroacetabular* or femoro-acetabular* or "femoroacetabular impingement" or FAIS or FAI).mp
3	Combine FAI Terms	1 or 2
4	Arthroscopy (MeSH)	"Arthroscopy"/
5	Arthroscopy (Keywords)	arthroscop*.mp
6	Combine Arthroscopy Terms	4 or 5
7	Study Types (MeSH and Publication Types)	"Cohort Studies"/ or "Randomized Controlled Trial".pt or "Controlled Clinical Trial".pt
8	Study Types (Keywords)	(RCT or randomised or randomized or cohort*).mp
9	Combining Study Type Terms	7 or 8
10	Date Restriction	2005/1/1:3000/12/12[pdat]
11	Animal Study Exclusion	("Animals"/ not "Humans"/)
12	Final Combination	3 and 6 and 9 and 10, not 11

Complete search strategy used for MEDLINE, including MeSH terms, keywords, Boolean operators, and date/eligibility filters. Search conducted on 11 October 2024.

Supplementary Table 2. Embase

Line	Concept	Search Terms
1	Femoroacetabular Impingement	('femoroacetabular*' OR 'fais':ti,ab,kw OR 'fai':ti,ab,kw OR 'femoroacetabular impingement'/exp OR 'femoroacetabular impingement' OR 'femoro-acetabular*' OR (('femoroacetabular' NEAR/3 'impingement'):ti,ab,kw))
2	Cohort Studies (EMBASE Terms)	'cohort analysis'/exp
3	Controlled Clinical Trials	'controlled clinical trial'/exp
4	Study Types (Keywords)	'rct':ti,ab,kw OR 'randomised':ti,ab,kw OR 'randomized':ti,ab,kw OR cohort*:ti,ab,kw
5	Combining Study Type Terms	2 OR 3 OR 4
6	Arthroscopy	'arthroscop*' OR 'arthroscopy'/exp OR 'arthroscopy'
7	Combine FAI, Arthroscopy, and Study Types	1 AND 5 AND 6
8	Date Restriction	[2005-2024]/py
9	Apply Date Restriction	7 AND 8
10	Animal Study Exclusion	'animal'/exp NOT 'human'/exp
11	Exclude Animal-Only Studies	9 NOT 10
12	Conference Abstract Exclusion	11 NOT 'conference abstract'/it

Complete Embase search syntax, including Emtree terms, keyword variations, and filters used to exclude animal-only studies and conference abstracts. Search conducted on 11 October 2024.

Supplementary Table 3. PEDro

Line	Concept	Search Terms and Filters
1	Femoroacetabular Impingement (Keyword)	femoroacetabular (Abstract and Title)

Search strategy for SPORTDiscus, detailing all keyword combinations, subject headings, and publication date limits. Search conducted on 11 October 2024.

Supplementary Table 4. SPORTDiscus

Line	Concept	Search Terms
1	Femoroacetabular Impingement (Base)	femoroacetabular impingement
2	Femoroacetabular Impingement (Keywords)	femoroacetabular* OR (femoroacetabular impingement OR fai) OR 'FAIS'
3	Femoroacetabular Impingement (Title/Abstract)	femoroacetabular* OR (femoroacetabular impingement OR fai) OR TI 'FAIS' OR AB 'FAIS'
4	Arthroscopy (Subject Heading)	DE "ARTHROSCOPY"
5	Arthroscopy (Keywords)	DE "ARTHROSCOPY" OR arthroscop*
6	Combine FAI and Arthroscopy	3 AND 5
7	Study Types (Subject Heading)	DE "COHORT analysis"
8	Study Types (Keywords)	DE "COHORT analysis" OR cohort* OR (randomized controlled trials OR rct OR randomised control trials) OR controlled clinical trial
9	Combine Study Type Terms	7 OR 7 OR 8
10	Combine FAI, Arthroscopy, and Study Types	6 AND 9
11	Date Restriction	Limiters - Publication Date: 20050101-
12	Final Search	10 AND 11

PEDro search terms and filters used to identify prospective studies of hip arthroscopy for FAIS. Search conducted on 11 October 2024.

Supplementary Table 5. Cochrane (CENTRAL)

Line	Concept	Search Terms
1	Femoroacetabular Impingement (MeSH)	MeSH descriptor: [Femoracetabular Impingement] explode all trees
2	Femoroacetabular Impingement (Keywords)	(Femoroacetabular* OR 'FAI' OR 'FAIS'):ti,ab,kw (Word variations have been searched)
3	Combine FAI Terms	1 OR 2
4	Arthroscopy (MeSH)	MeSH descriptor: [Arthroscopy] explode all trees
5	Arthroscopy (Keywords)	(arthroscop*):ti,ab,kw (Word variations have been searched)
6	Combine Arthroscopy Terms	4 OR 5
7	Combine FAI and Arthroscopy with Date Restriction	3 AND 6 with Cochrane Library publication date from Jan 2005 to present

Cochrane CENTRAL strategy including MeSH terms, keyword variants, and date restrictions. Search conducted on 11 October 2024.

Supplementary treatment

Supplementary Table 6. Summary of the harms of hip arthroscopy among patients with FAIS, including unweighted proportions, in studies reporting revision surgery, and the reason for revision surgery.

Complication	Studies, n	Events, n	Hips, n[a]	Unweighted Proportion (95% CI)
**Reason for revision surgery**	23	171	2170	7.9% (6.8-9.1)
Adhesions	14	62	1356	4.6% (3.5–5.8)
Labral pathology[b]	15	52	1369	3.8% (2.9–5.0)
Residual Impingement	15	29	1691	1.7% (1.2–2.5)
Capsular defects[c]	7	13	760	1.7% (0.9–2.9)
Psoastendinopathy	3	5	263	1.9% (0.6–4.4)
Symptomatic heterotopic ossification	2	3	210	1.4% (0.3–4.1)
Cartilage injury	2	2	291	0.7% (0.1–2.5)
Recurrent pain	1	1	114	0.9% (0.0–4.8)
Osteoarthritis progression	1	1	57	1.8% (0.0–9.4)
Osteoid Osteoma	1	1	51	2.0% (0.1–10.5)
Subspine Impingement	1	1	84	1.2% (0.0–6.5)
Iliotibial band release	1	1	160	0.6% (0.0–3.4)

[a]Total hips reflect the full review sample for harms; fewer hips for some outcomes due to incomplete reporting in included studies.
[b]Labral pathology includes tears, refixation, debridement, calcification, or inflammation.
[c]Capsular defects include closure, plication, laxity, or thickening issues, distinct from adhesions.

Harms, irrespective of follow-up duration.

Supplementary Figure 1. Proportion of conversion to total hip arthroplasty following primary hip arthroscopy in patients with femoroacetabular impingement.

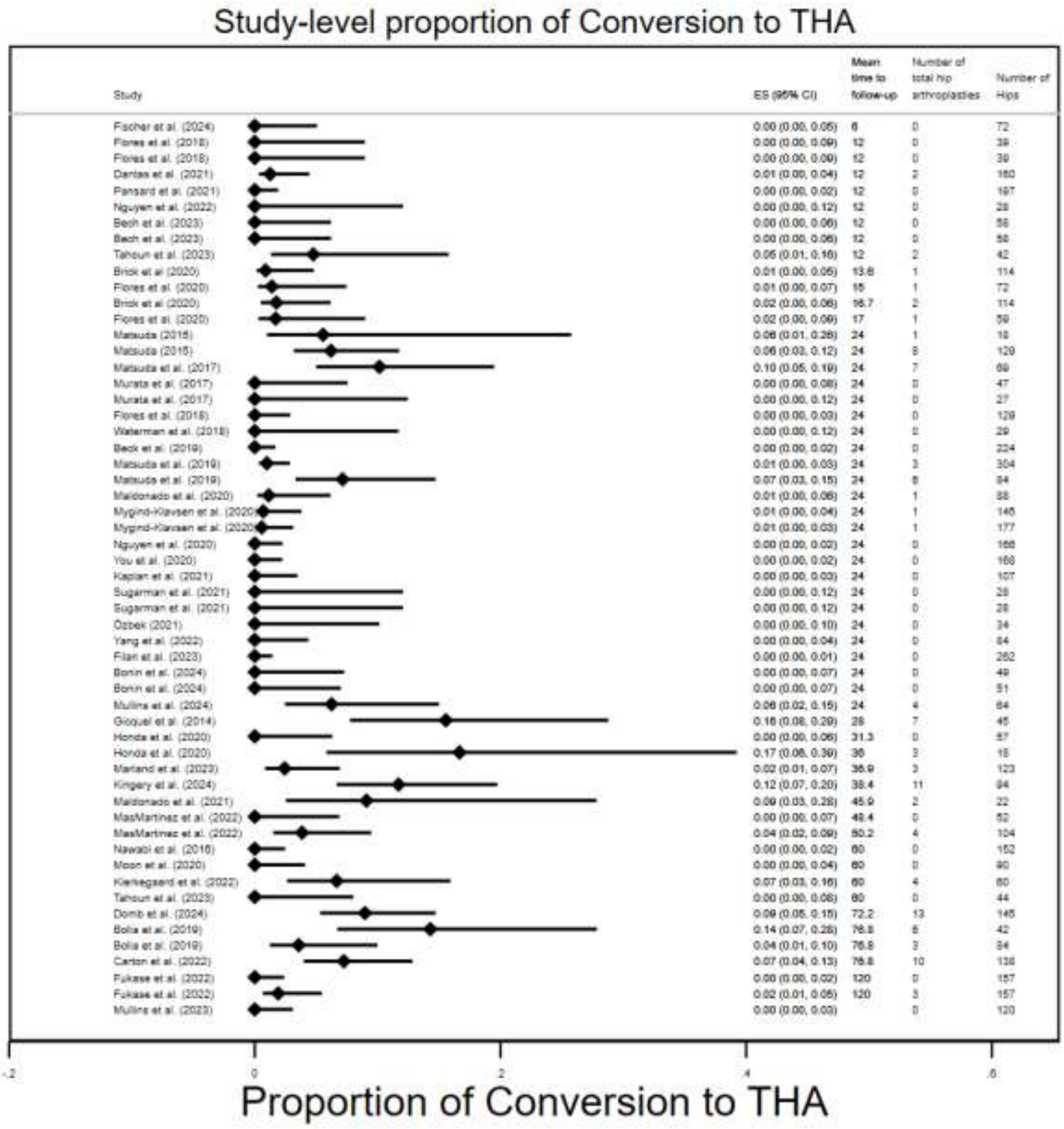

Supplementary Figure 2. Proportion of revision surgery for primary arthroscopy in patients with femoroacetabular impingement.

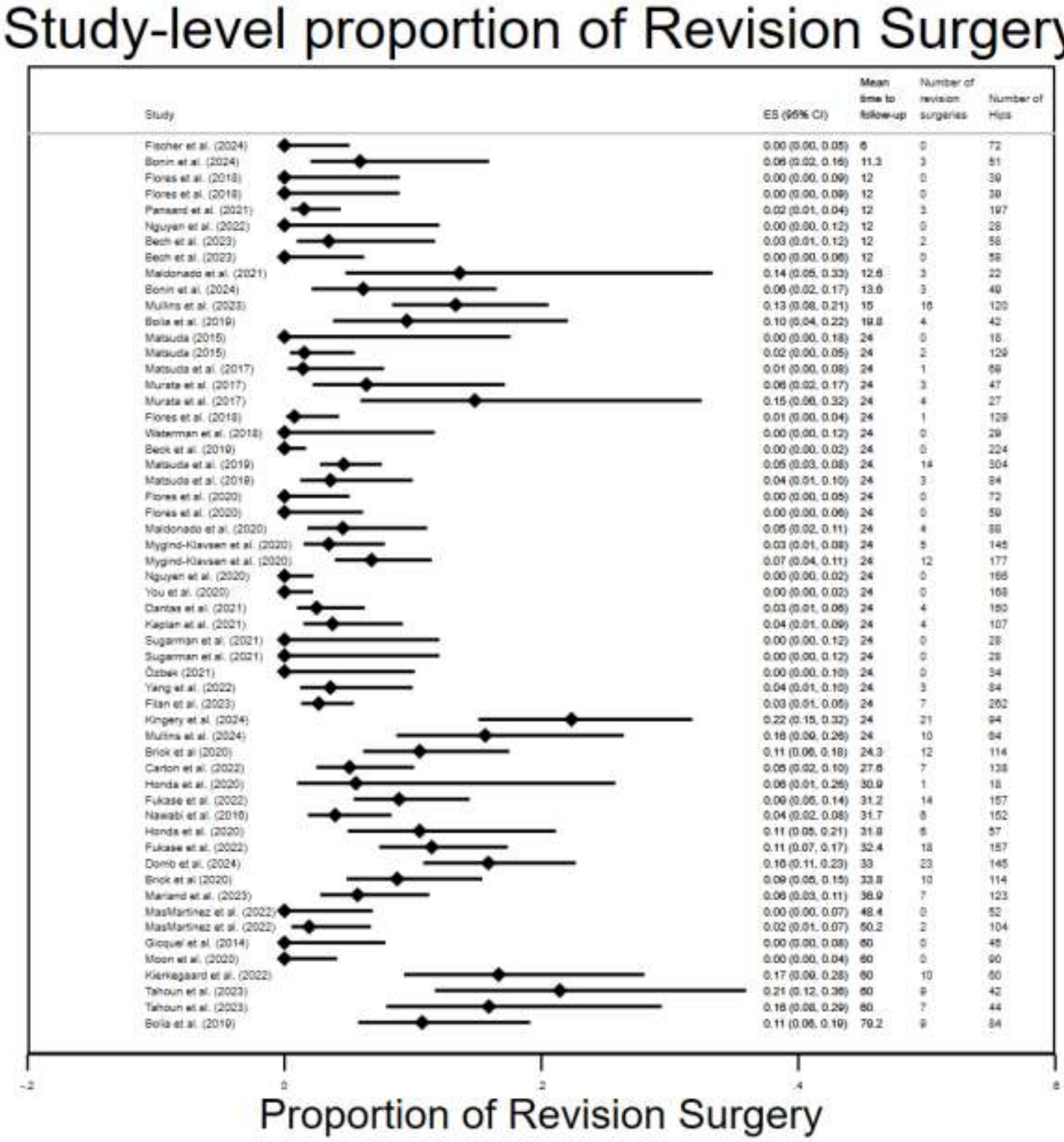

Supplementary Figure 3. Proportion of nerve paresthesia for primary hip arthroscopy in patients with FAIS, in studies reporting at least one such event.

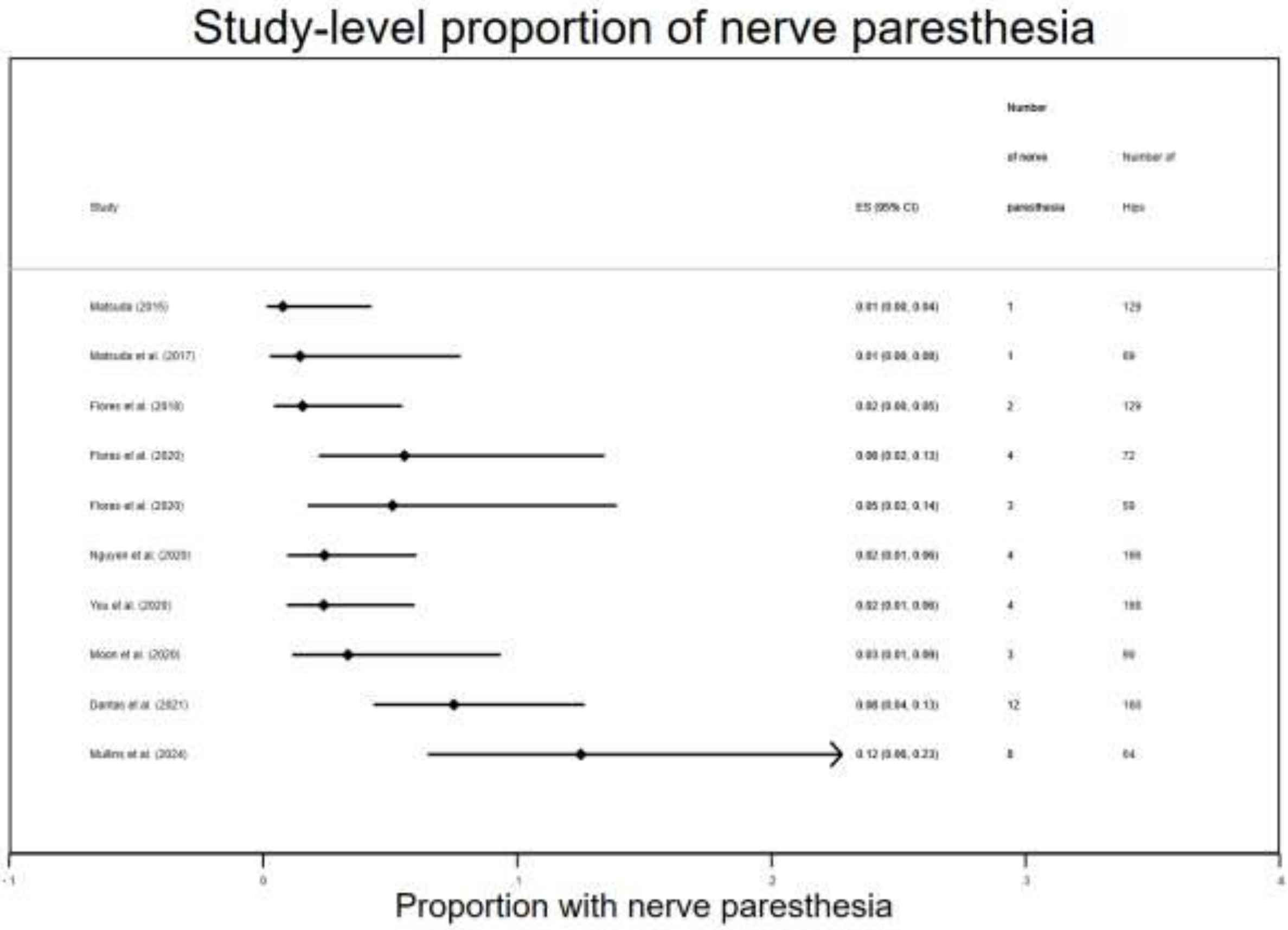

Supplementary Figure 4. Proportion of nerve paresthesia for primary hip arthroscopy in patients with FAIS.

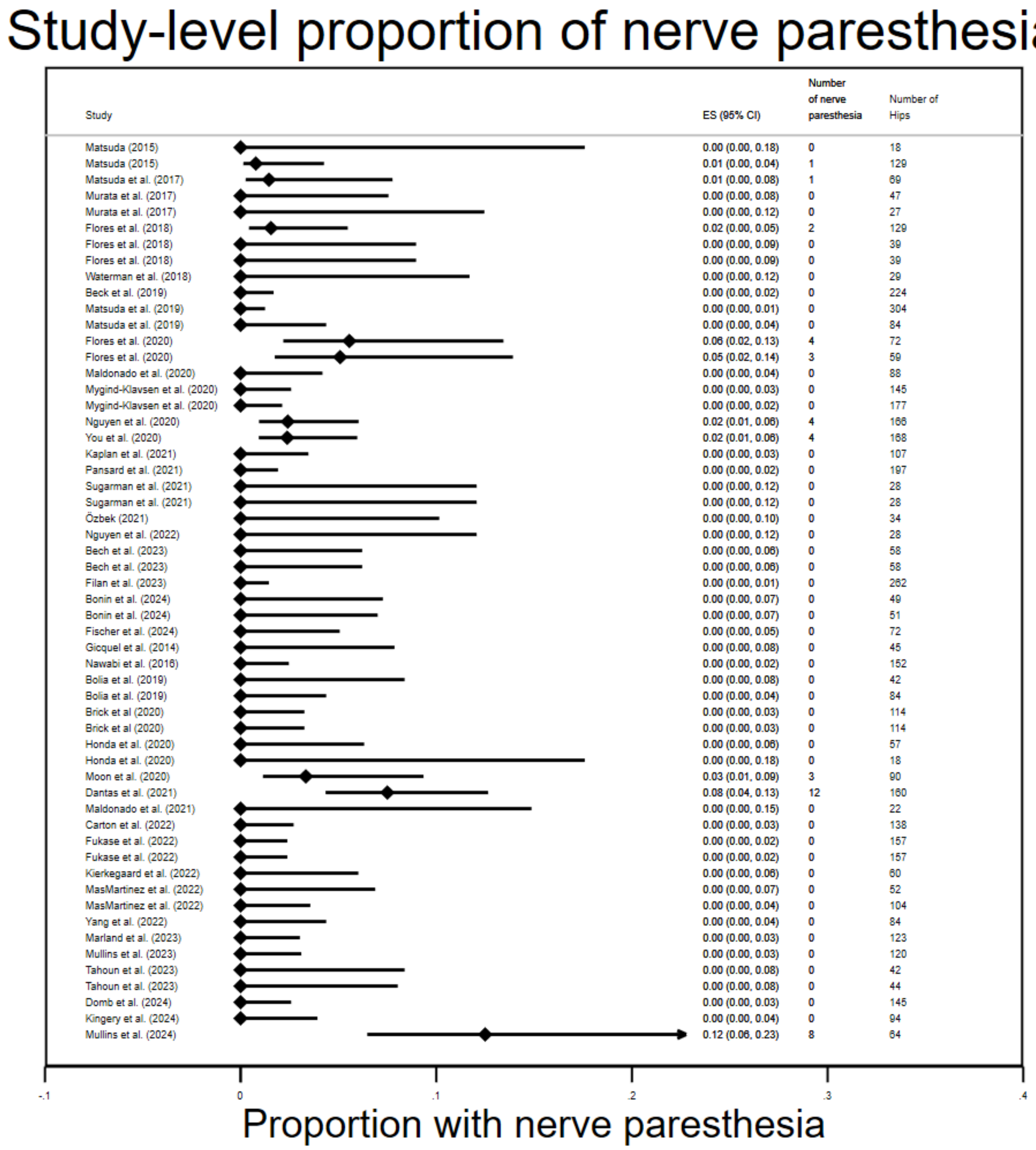

Harms, sub-analysis of follow-up duration

Supplementary Figure 5. Proportion of conversion to total hip arthroplasty for primary hip arthroscopy in patients with femoroacetabular impingement. Follow-up duration up to 2 years.

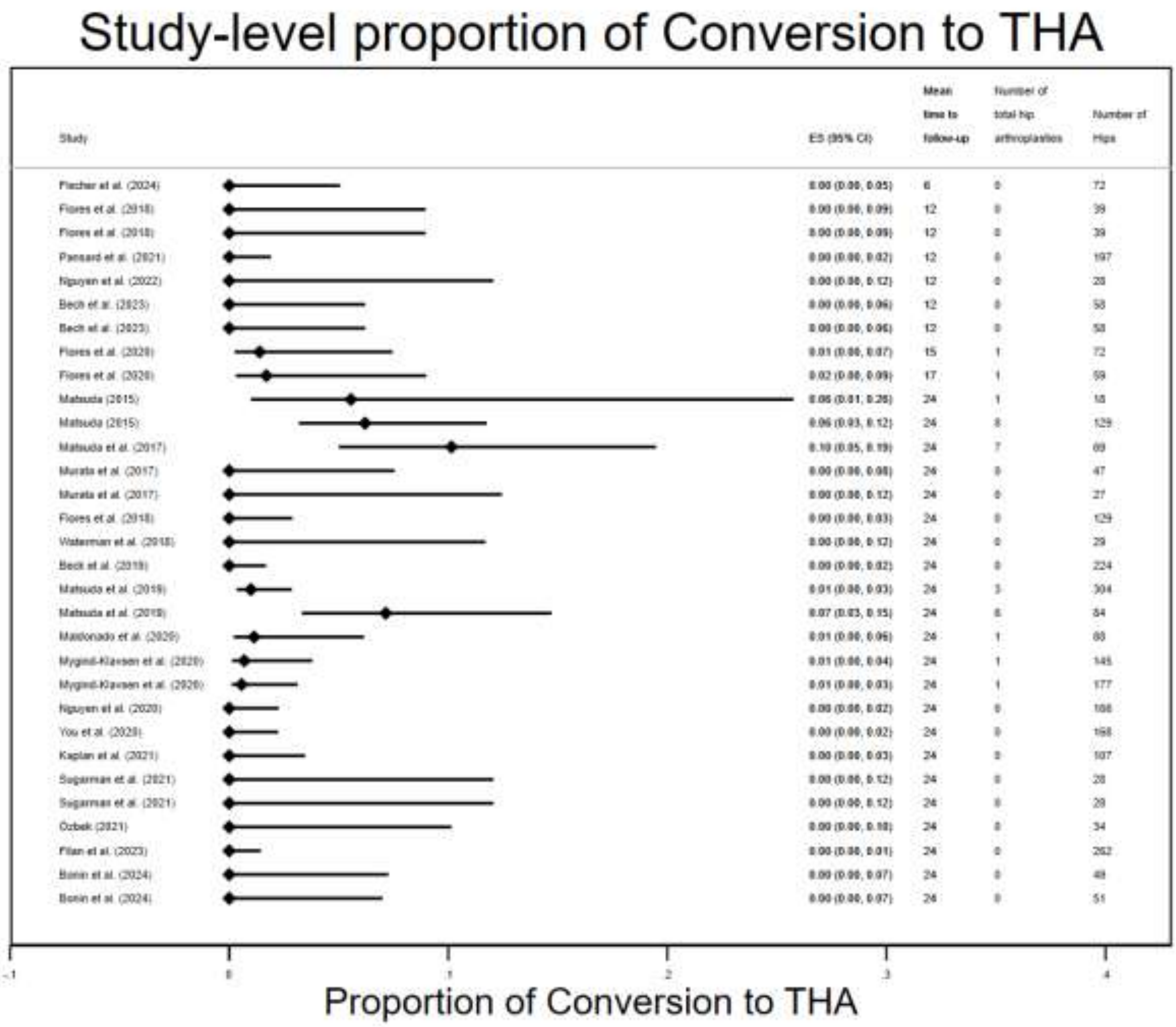

Supplementary Figure 6. Proportion of conversion to total hip arthroplasty for primary hip arthroscopy in patients with femoroacetabular impingement. Follow-up duration after 2 years.

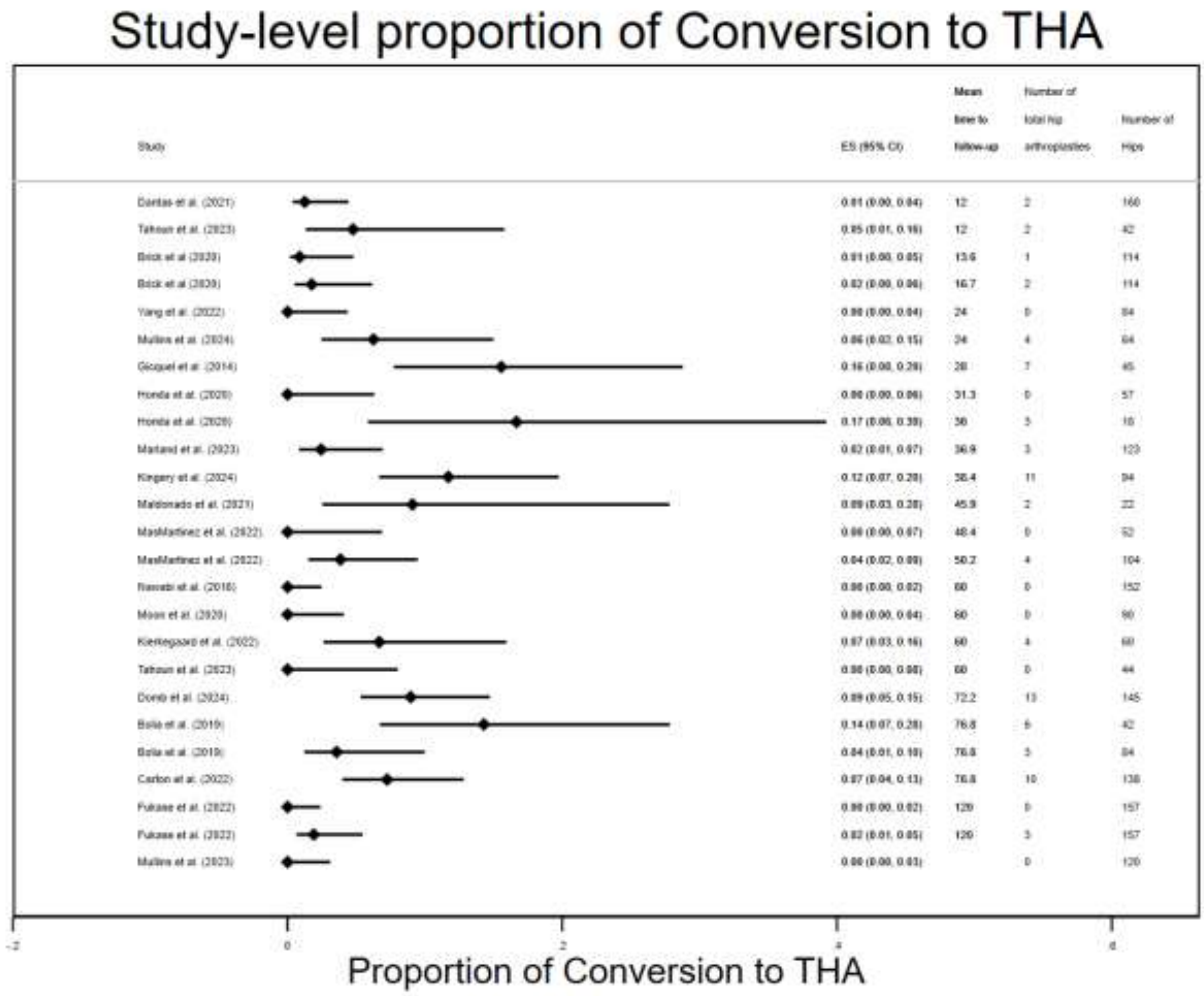

Supplementary Figure 7. Proportion of revision surgery for primary hip arthroscopy in patients with femoroacetabular impingement. Follow-up duration up to 2 years.

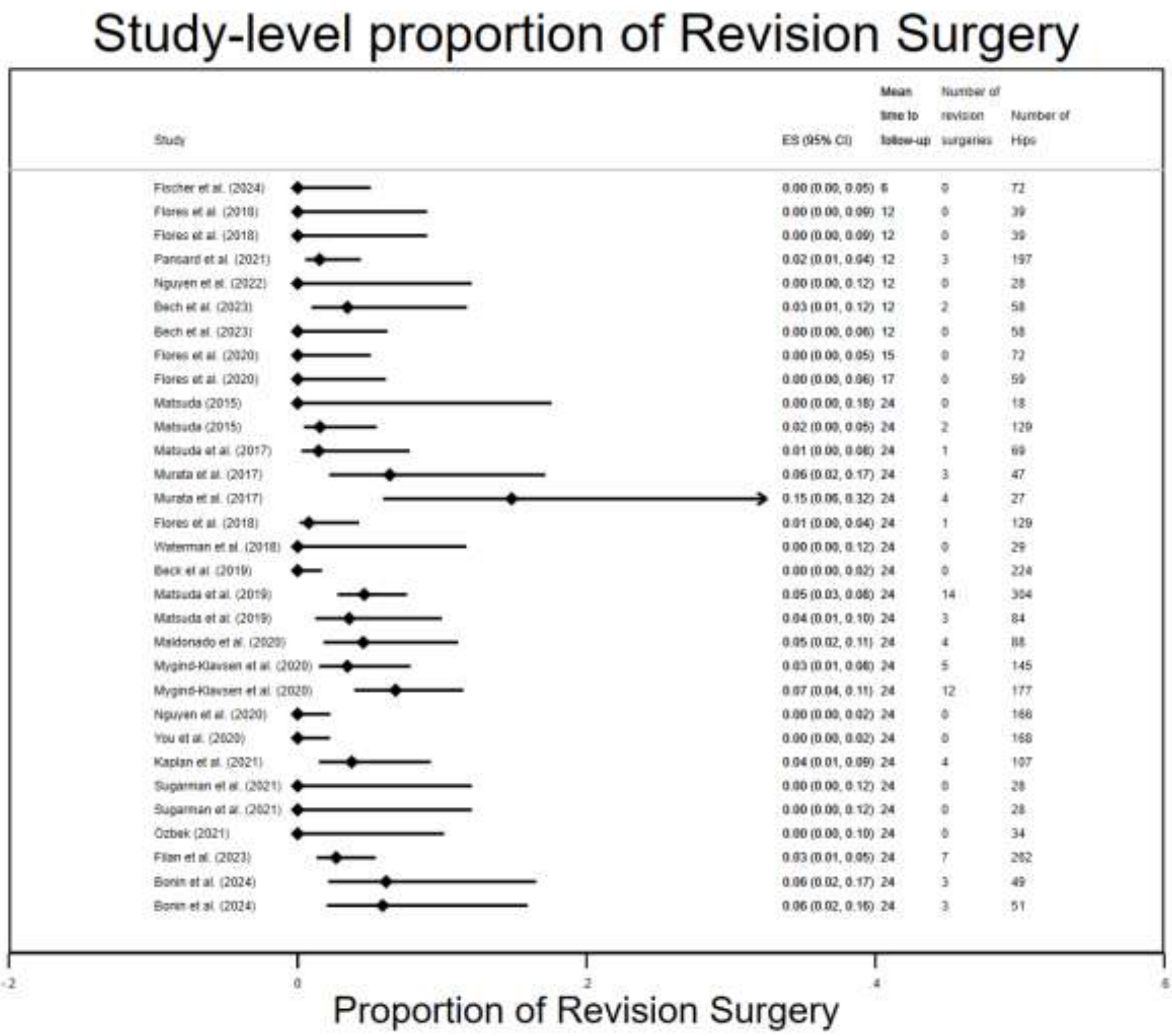

Supplementary Figure 8. Proportion of revision surgery for primary hip arthroscopy in patients with femoroacetabular impingement. Follow-up duration after 2 years.

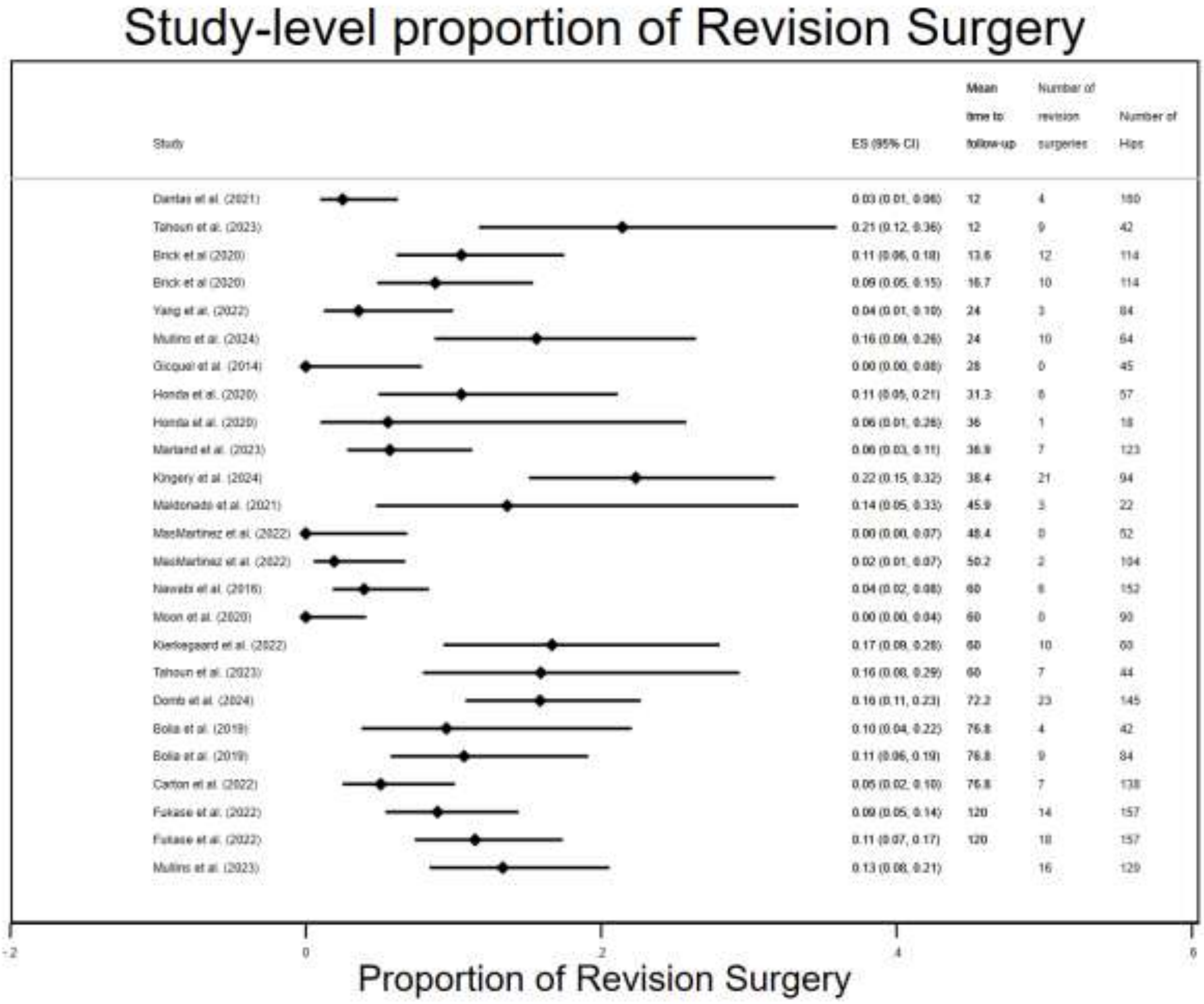

Harms, sub-analysis of capsular closure versus no capsular closure.

Supplementary Figure 9. Proportion of conversion to total hip arthroplasty for primary hip arthroscopy in patients with femoroacetabular impingement. Capsular repair.

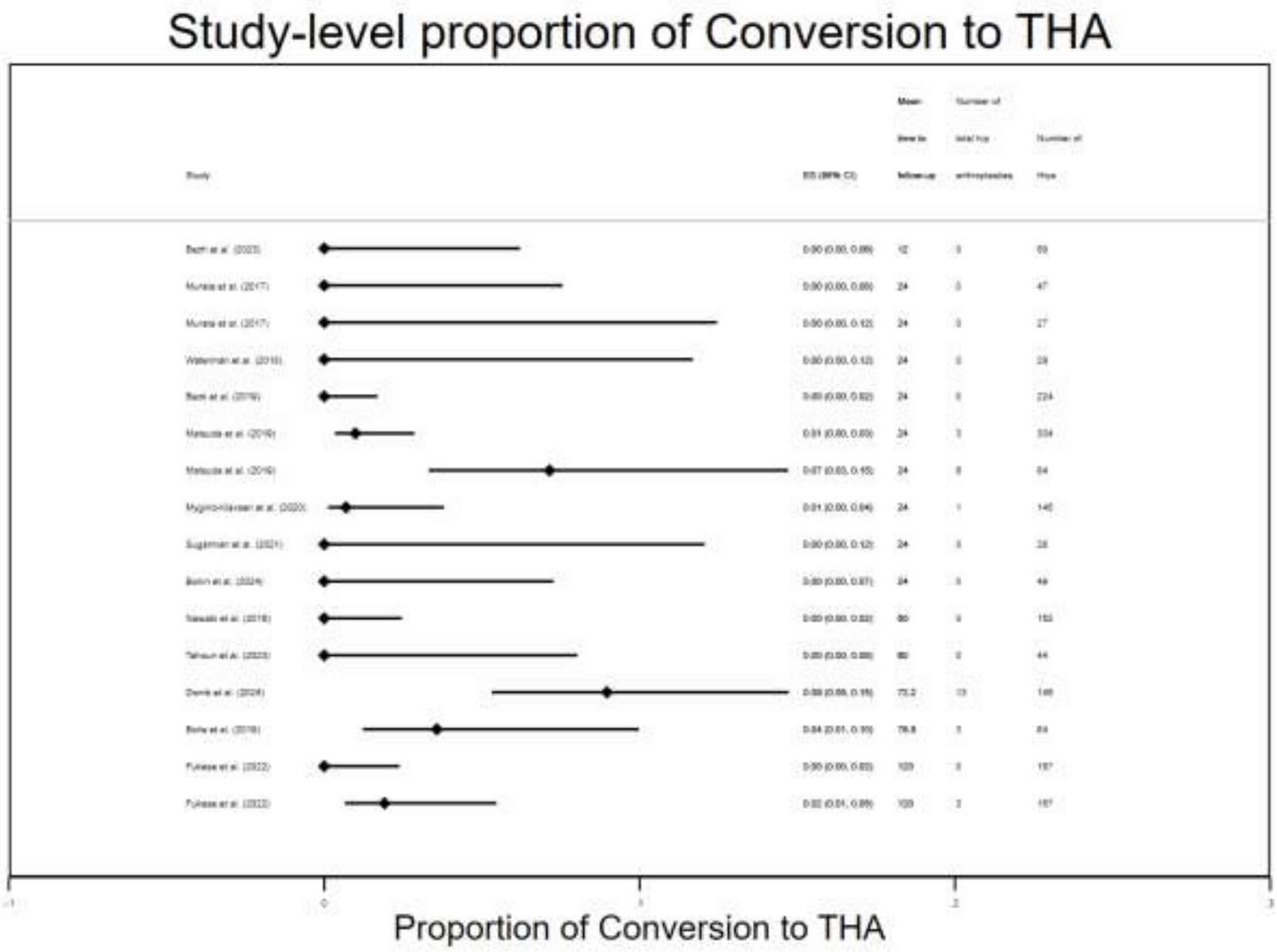

Supplementary Figure 10. Proportion of conversion to total hip arthroplasty for primary hip arthroscopy in patients with femoroacetabular impingement. No capsular repair.

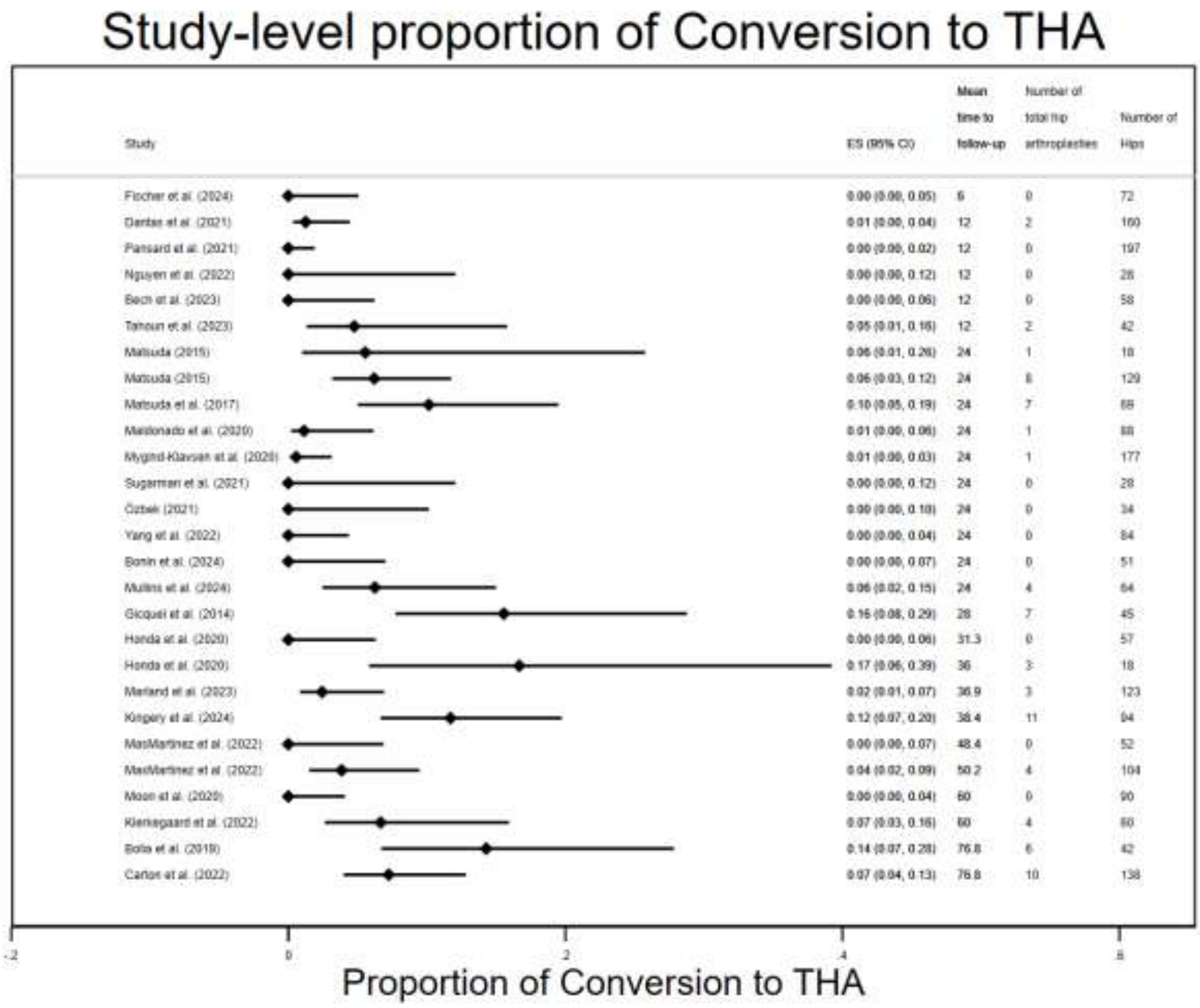

Supplementary Figure 11. Proportion of revision surgery for primary hip arthroscopy in patients with femoroacetabular impingement. Capsular repair.

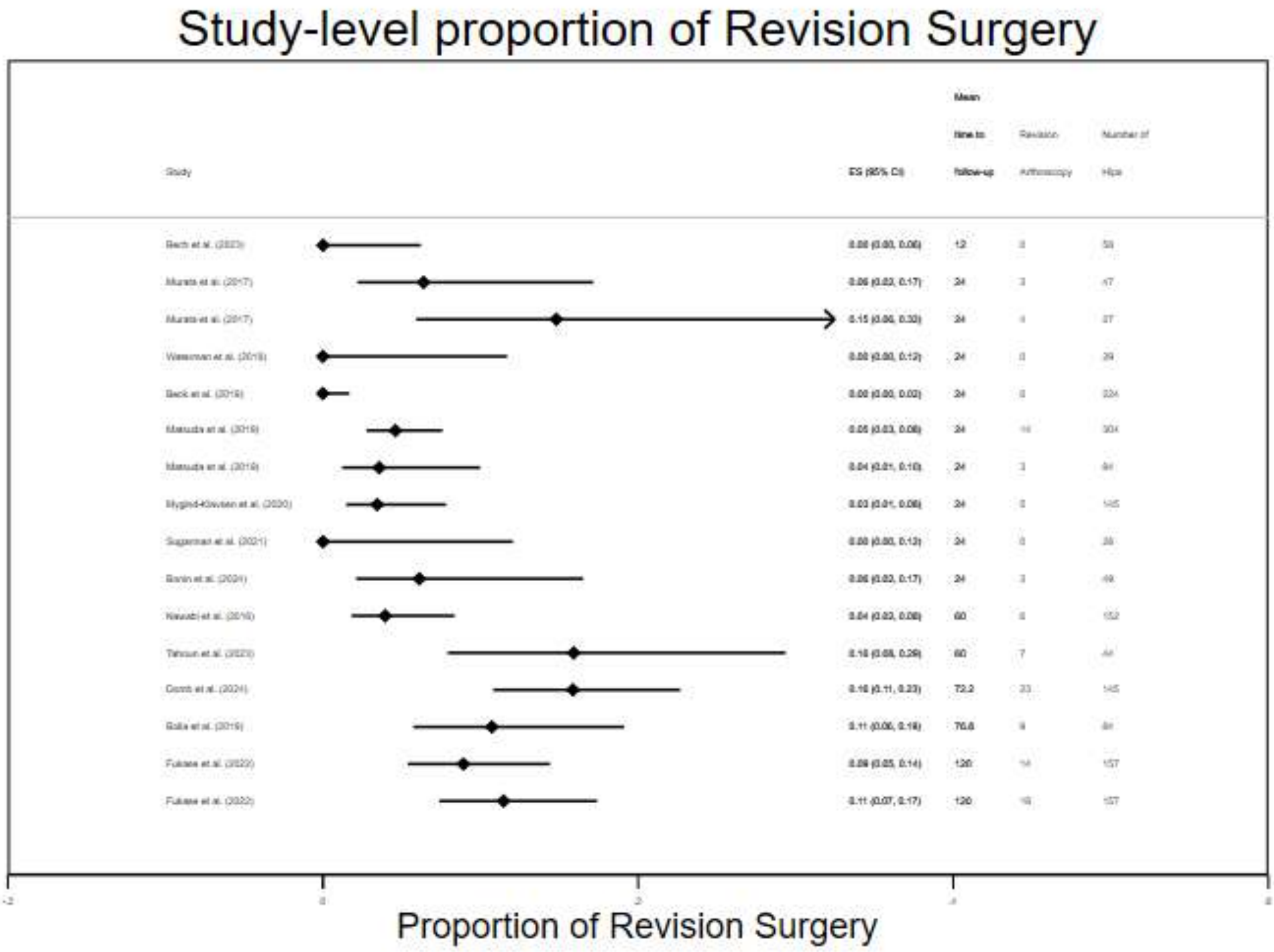

Supplementary Figure 12. Proportion of revision surgery for primary hip arthroscopy in patients with femoroacetabular impingement. No capsular repair.

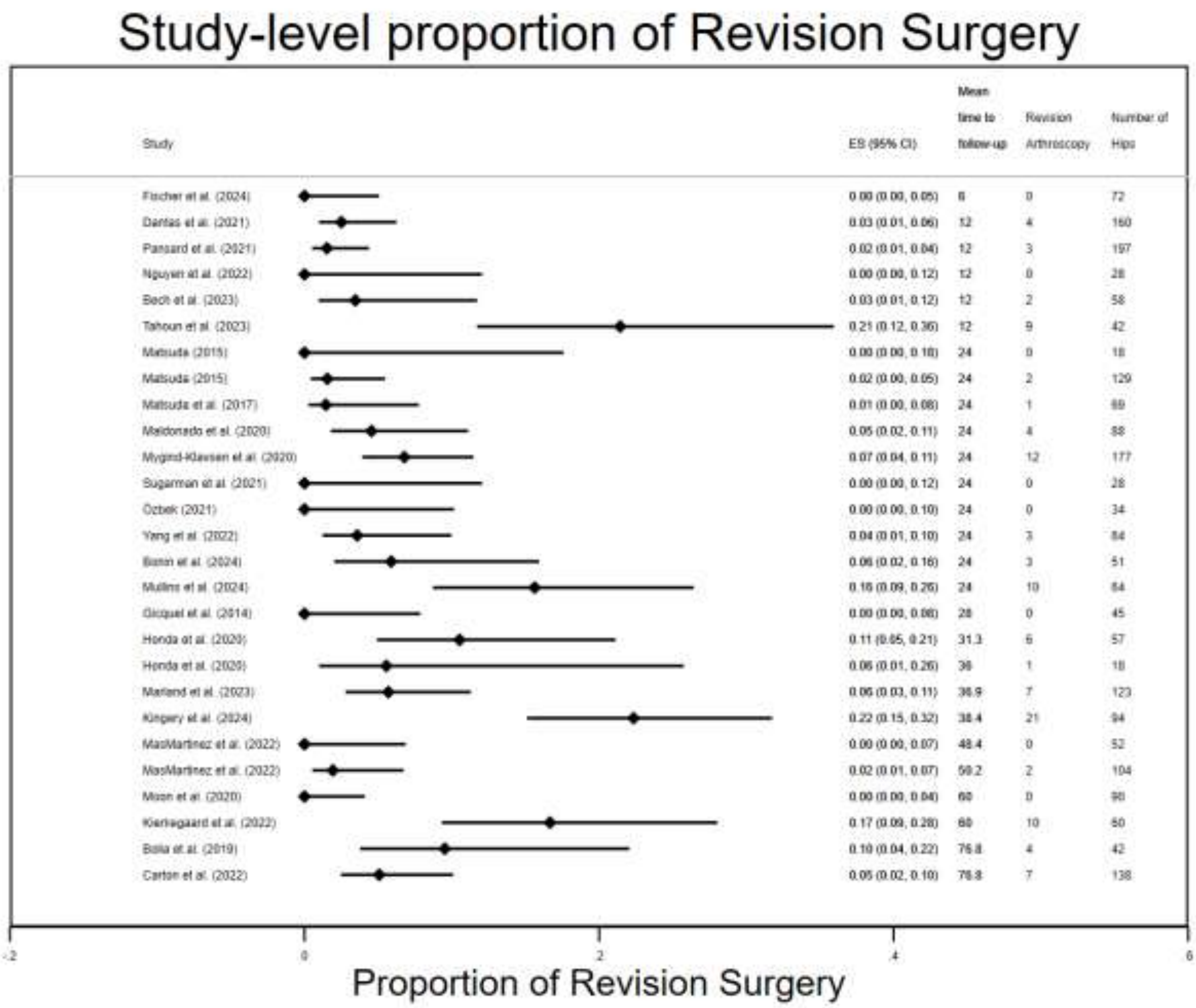

Benefits across hip-related outcomes, irrespective of follow-up duration.

Supplementary Figure 13. Change of Pain subscales from baseline to each follow-up, after primary hip arthroscopy for patients with femoroacetabular impingement.

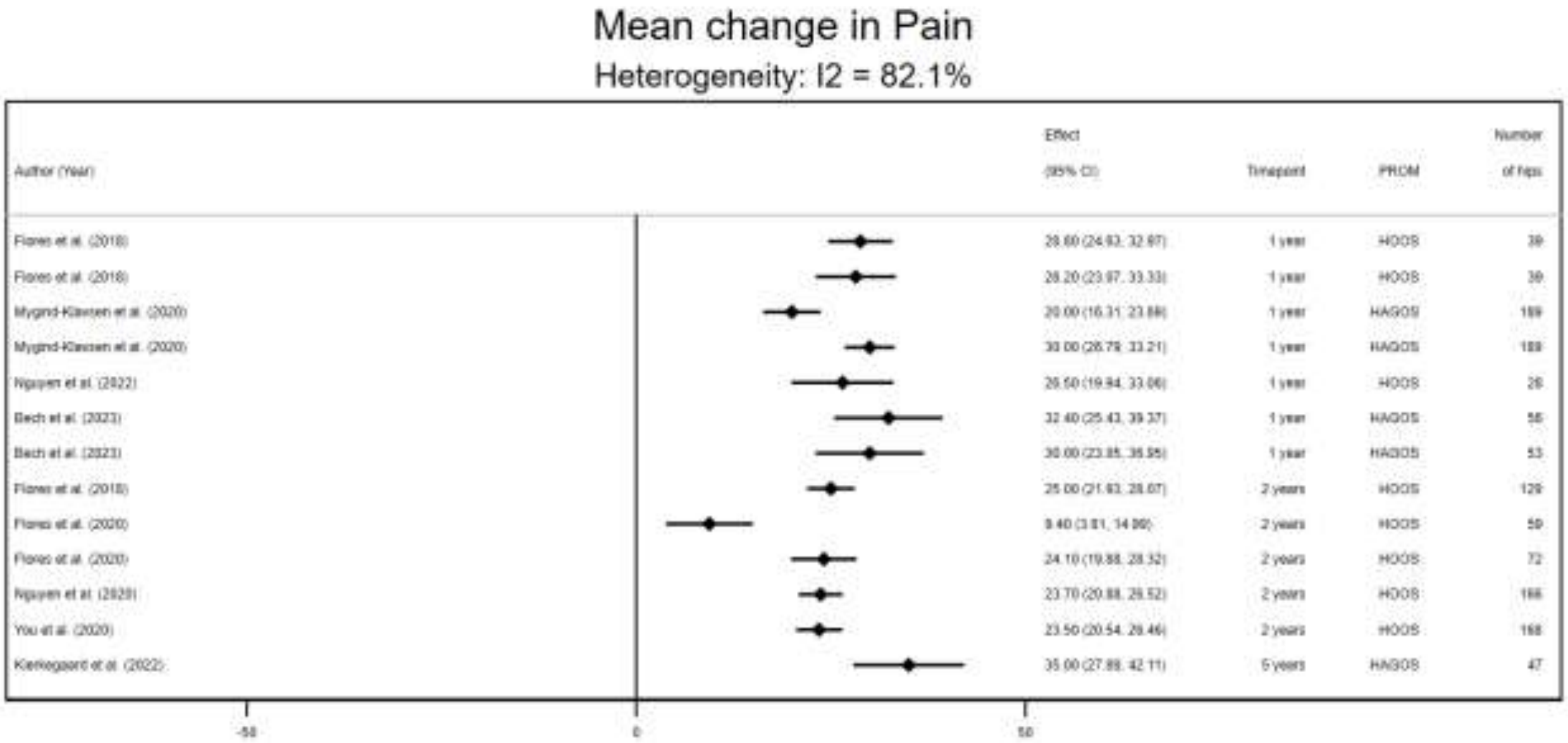

Supplementary Figure 14. Change in the Activities of Daily Living subscales from baseline to each follow-up, after primary hip arthroscopy for patients with femoroacetabular impingement.

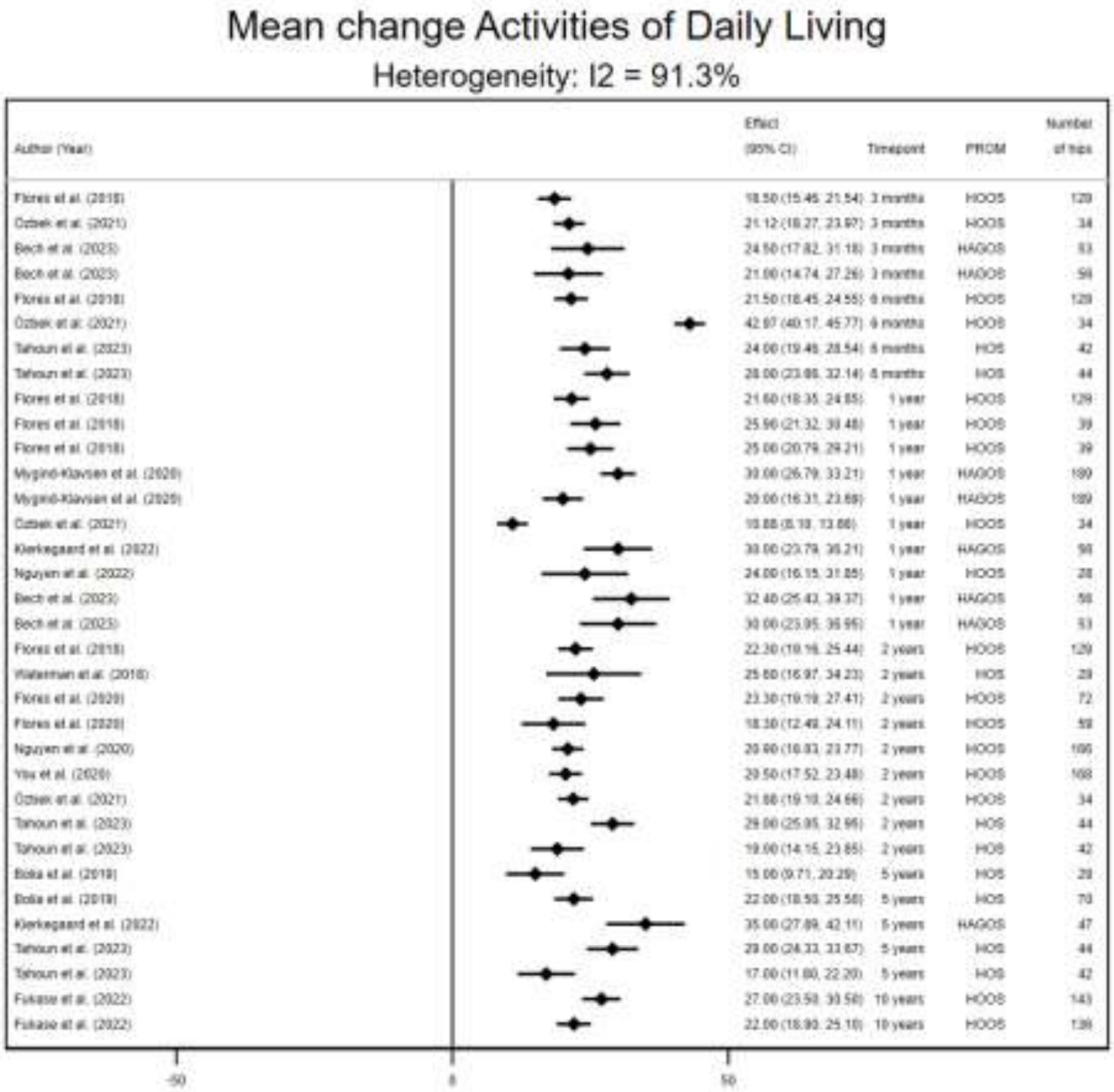

Supplementary Figure 15. Change in the Sports Participation subscales from baseline to each follow-up, after primary hip arthroscopy for patients with femoroacetabular impingement.

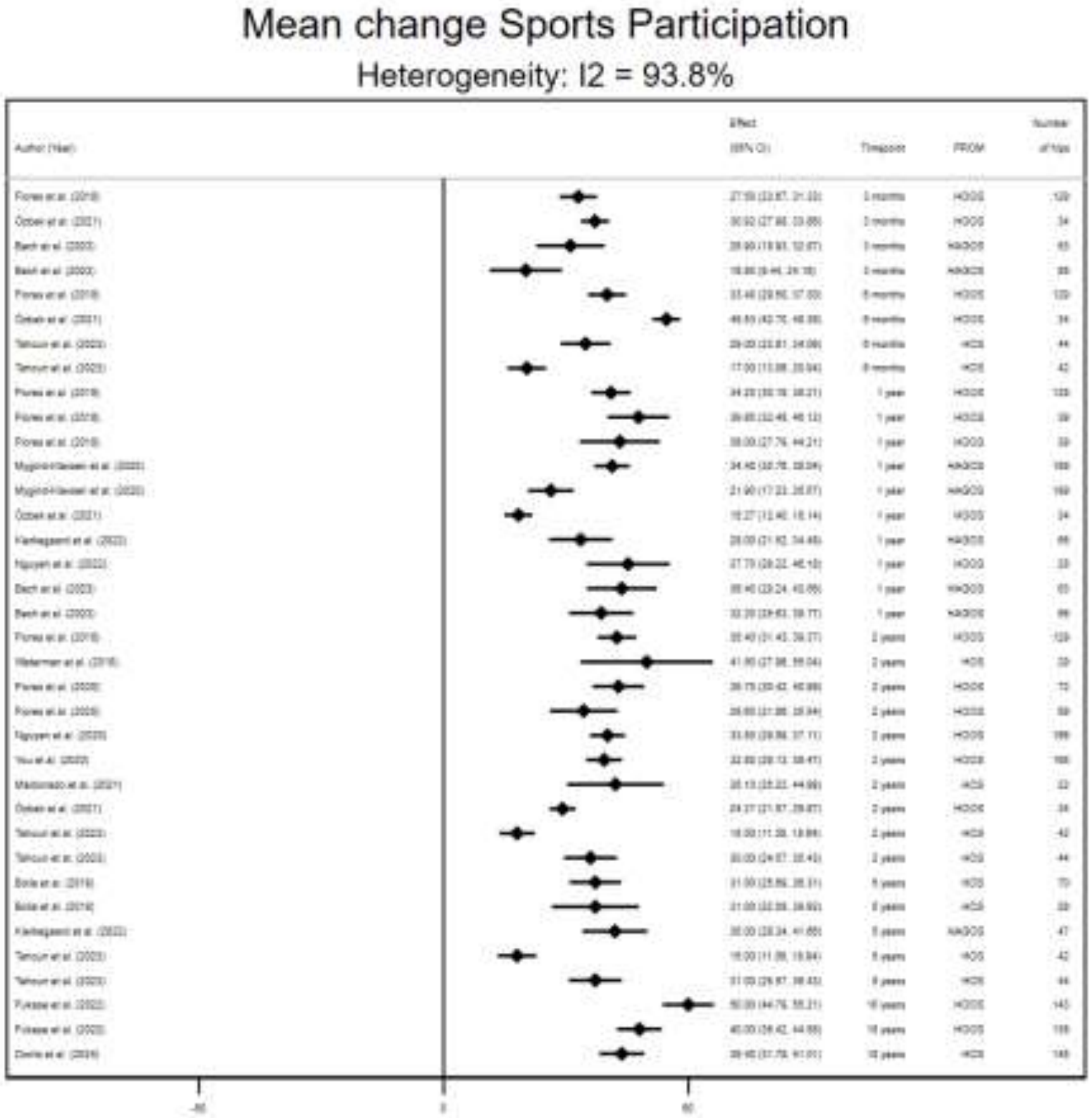

Supplementary Figure 16. Change in the Quality-of-Life subscales from baseline to each follow-up, after primary hip arthroscopy for patients with femoroacetabular impingement.

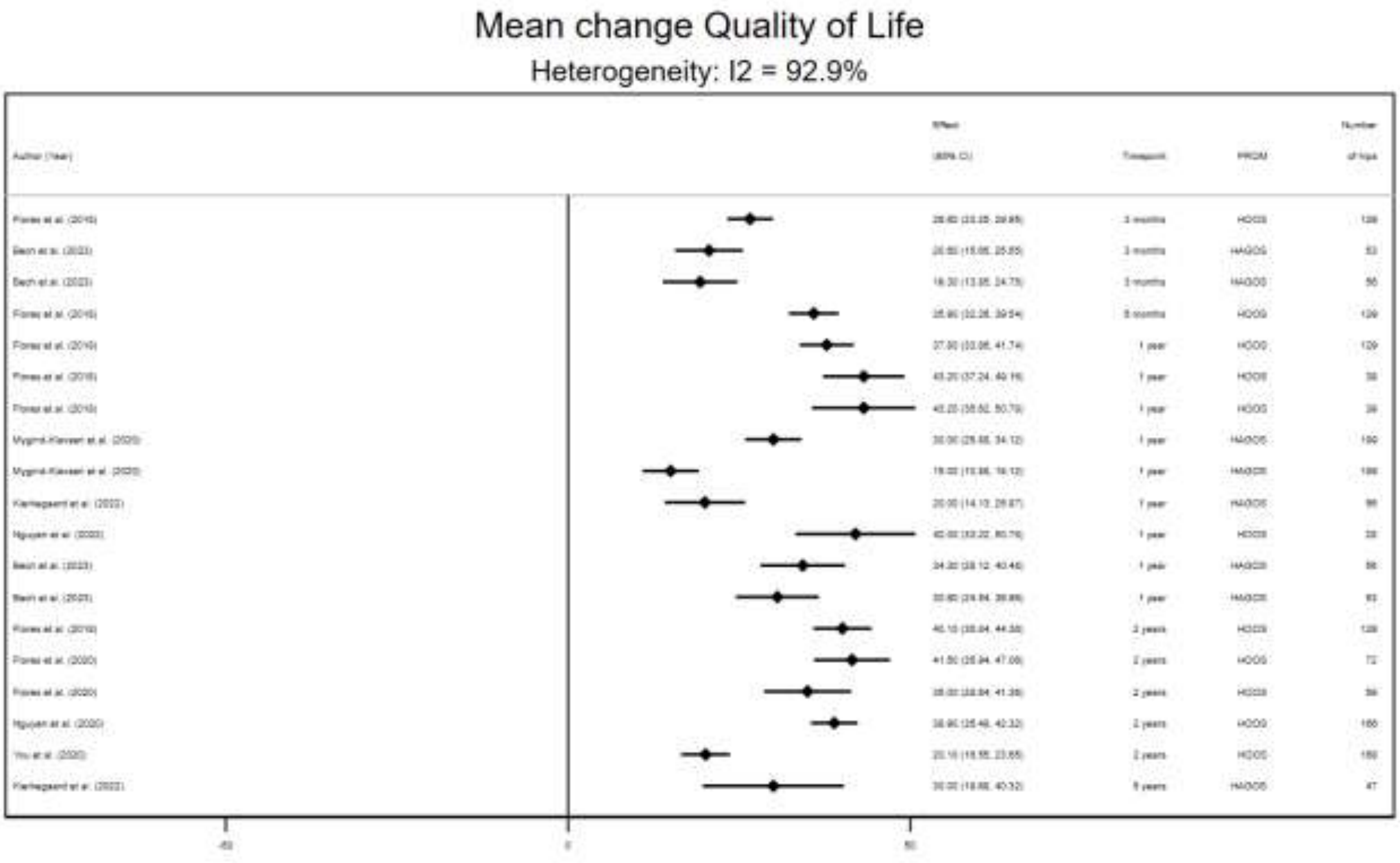

Supplementary Figure 17. Change in the Symptoms subscales from baseline to each follow-up, after primary hip arthroscopy for patients with femoroacetabular impingement.

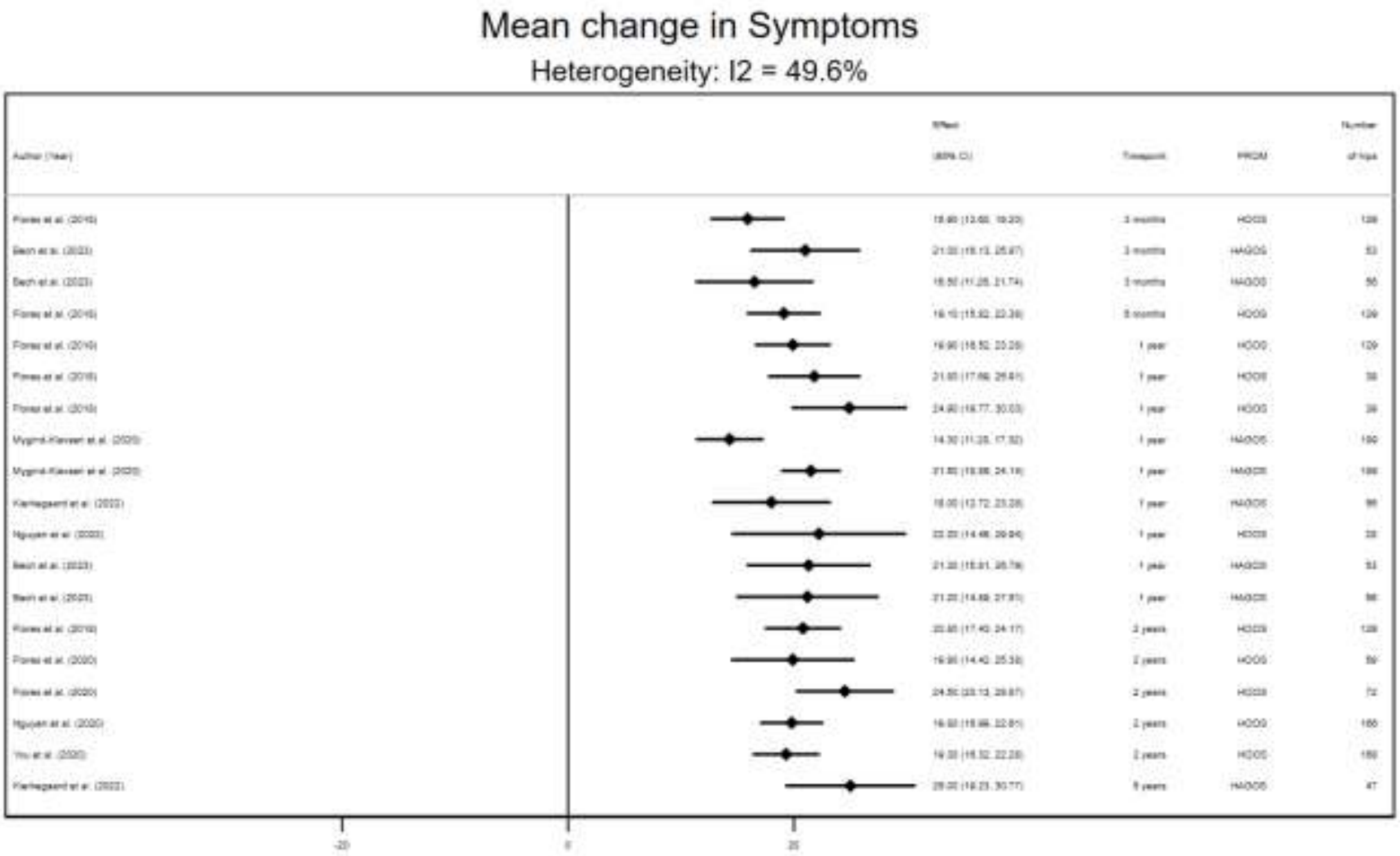

Supplementary Figure 18. Change in the composite Total scores from baseline to each follow-up, after primary hip arthroscopy for patients with femoroacetabular impingement.

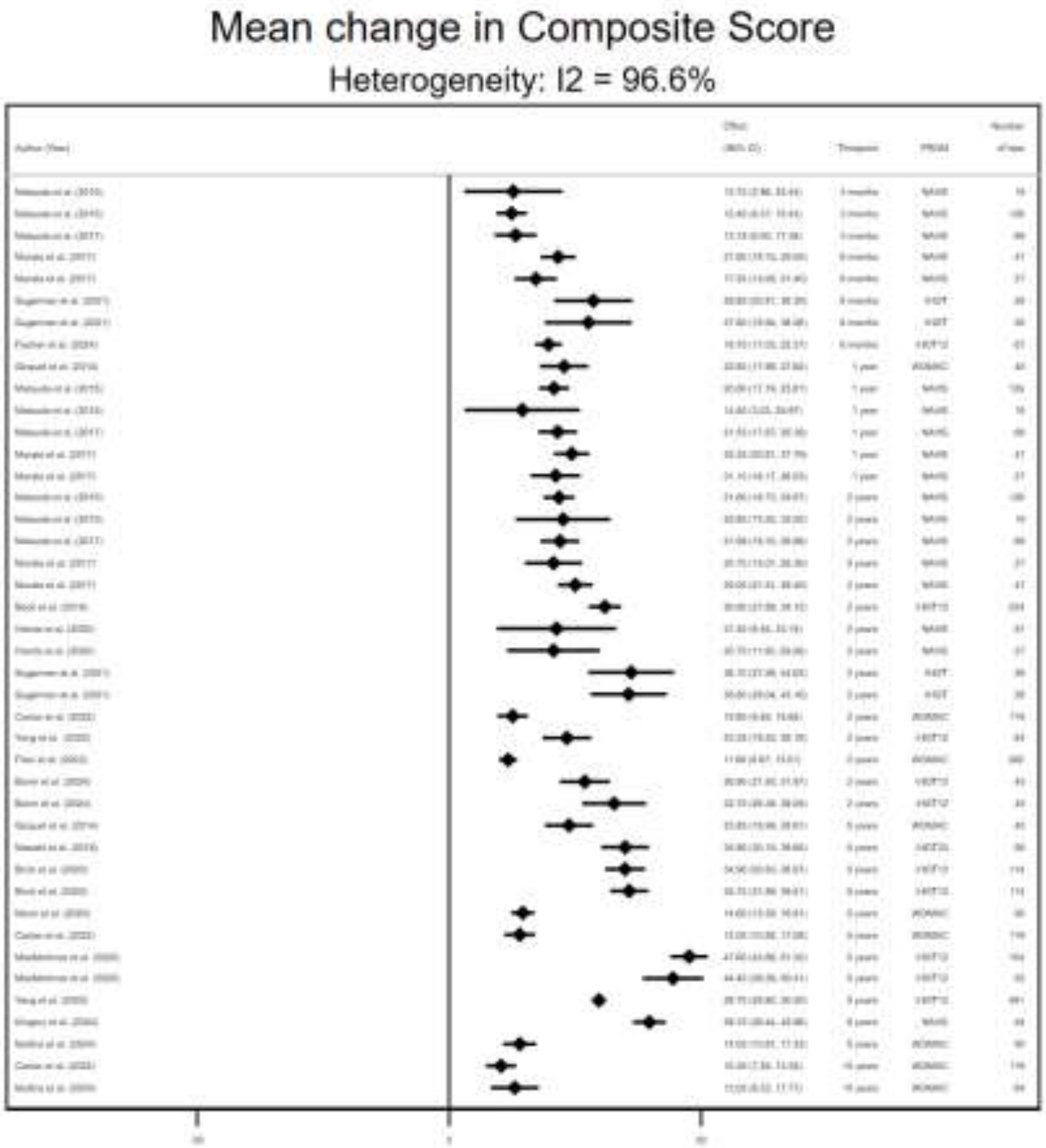

Benefits across hip-related outcomes, including a sub-analysis of follow-up durations of 3- and 6 months, 1-, 2-, 5-, and 10-year follow-ups.

Supplementary Figure 19. Change in the Pain subscales from baseline to each follow-up for primary hip arthroscopy in patients with femoroacetabular impingement.

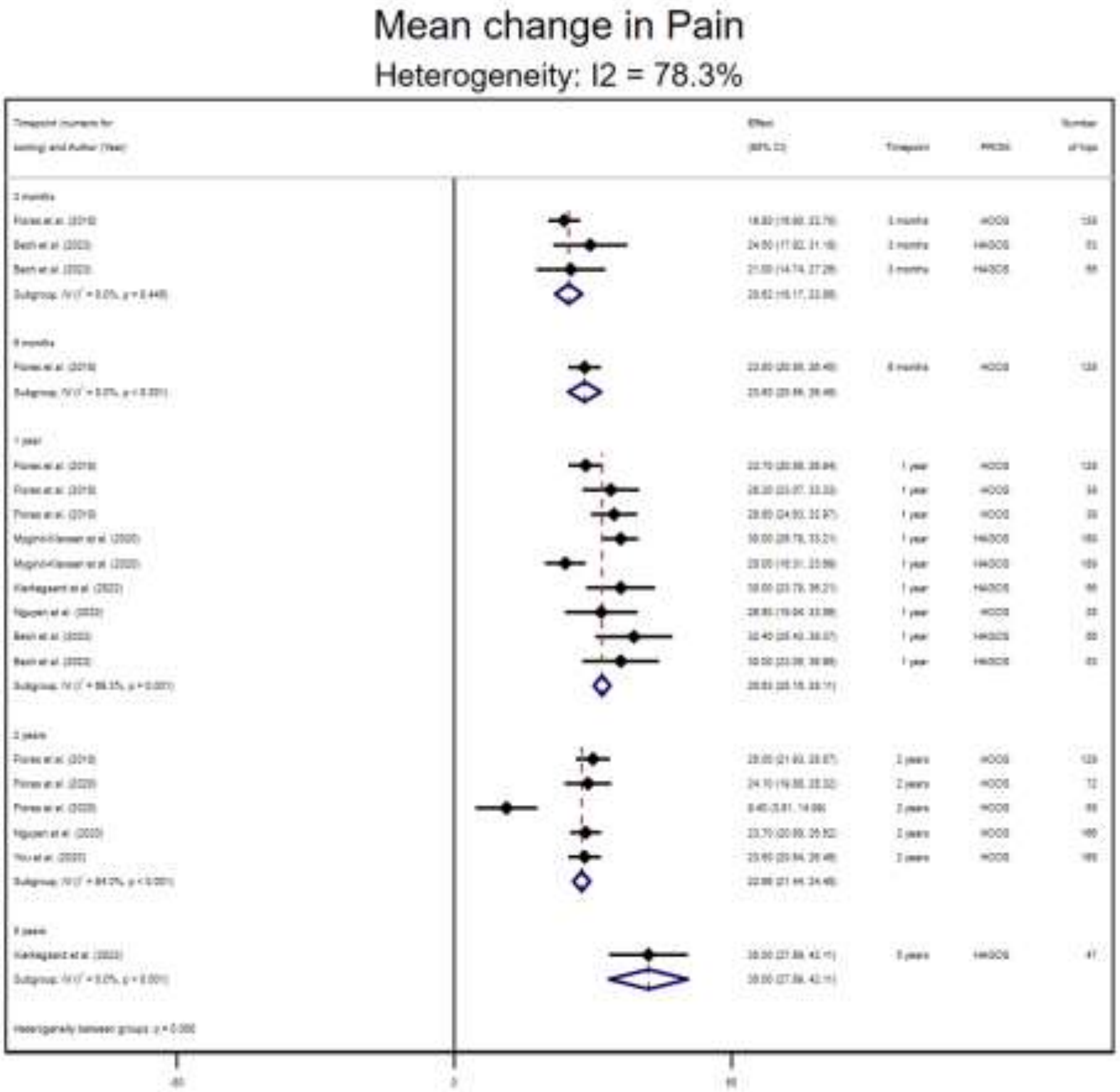

Supplementary Figure 20. Change in the Activities of Daily Living subscales from baseline to each follow-up, after primary hip arthroscopy in patients with femoroacetabular impingement.

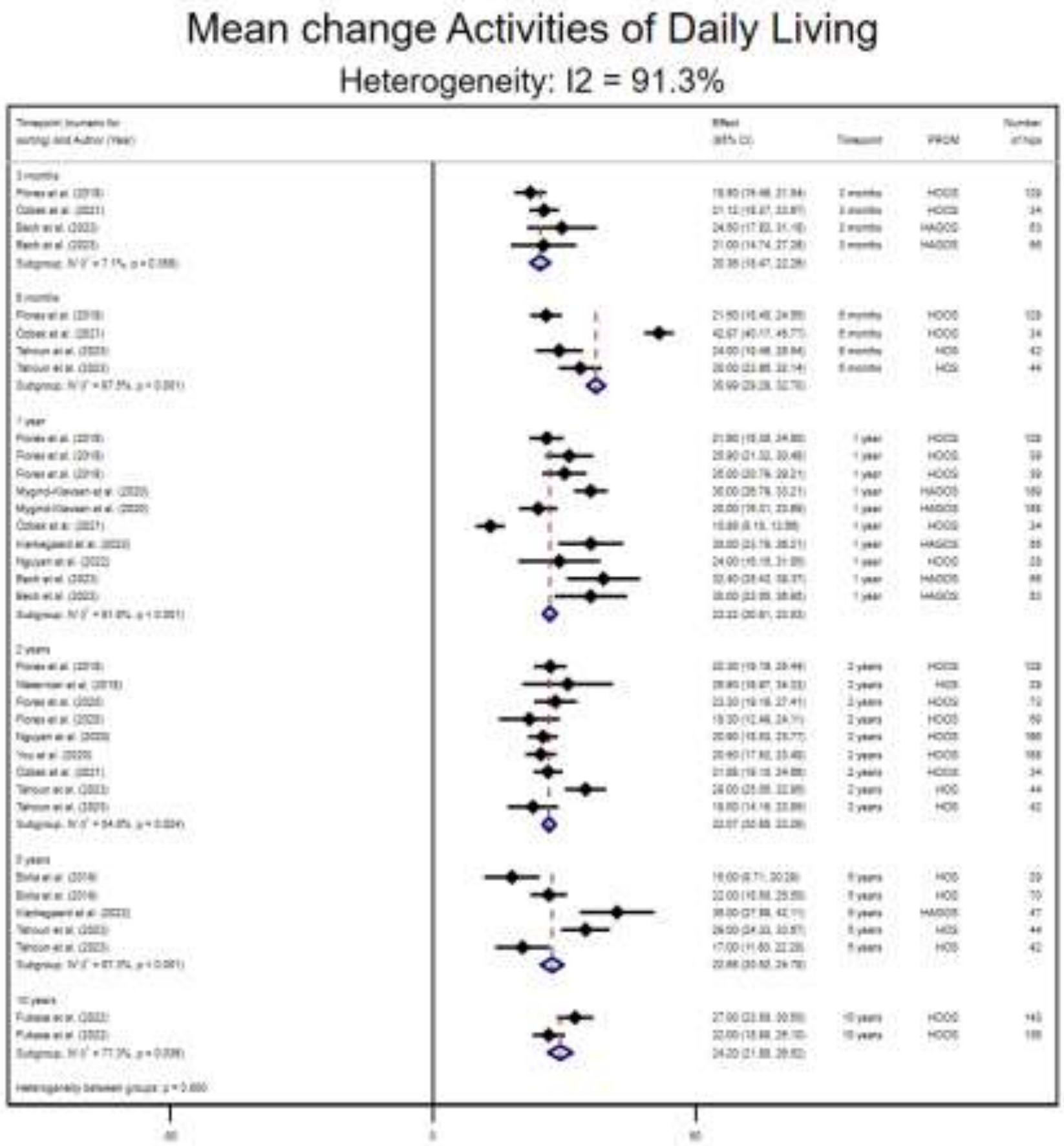

Supplementary Figure 21. Change in the Sports Participation subscales from baseline to each follow-up, after primary hip arthroscopy in patients with femoroacetabular impingement.

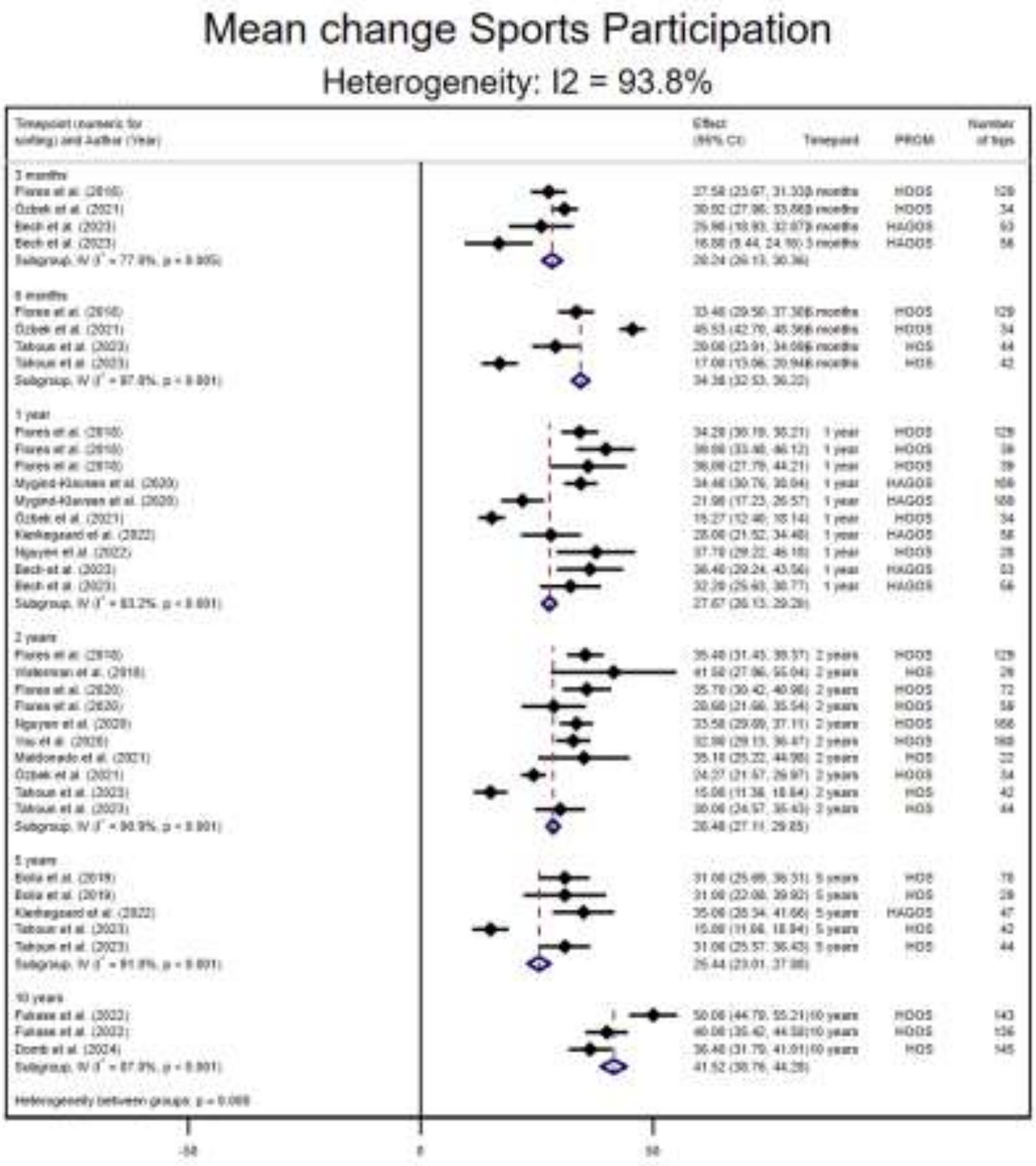

Supplementary Figure 22. Change in the Quality-of-Life subscales from baseline to each follow-up, after primary hip arthroscopy in patients with femoroacetabular impingement.

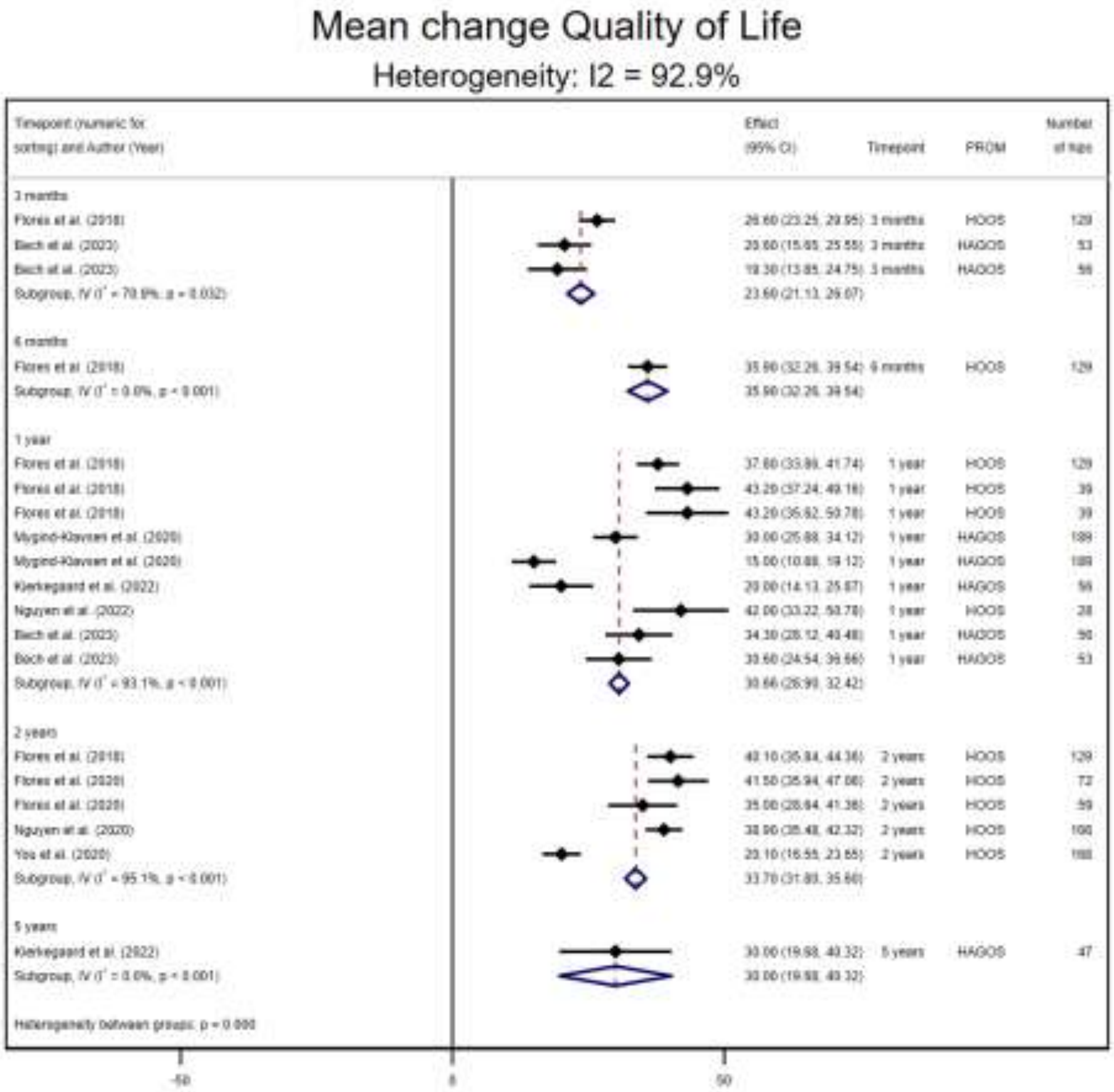

Supplementary Figure 23. Change in the Symptoms subscale from baseline to each follow-up, after primary hip arthroscopy in patients with femoroacetabular impingement.

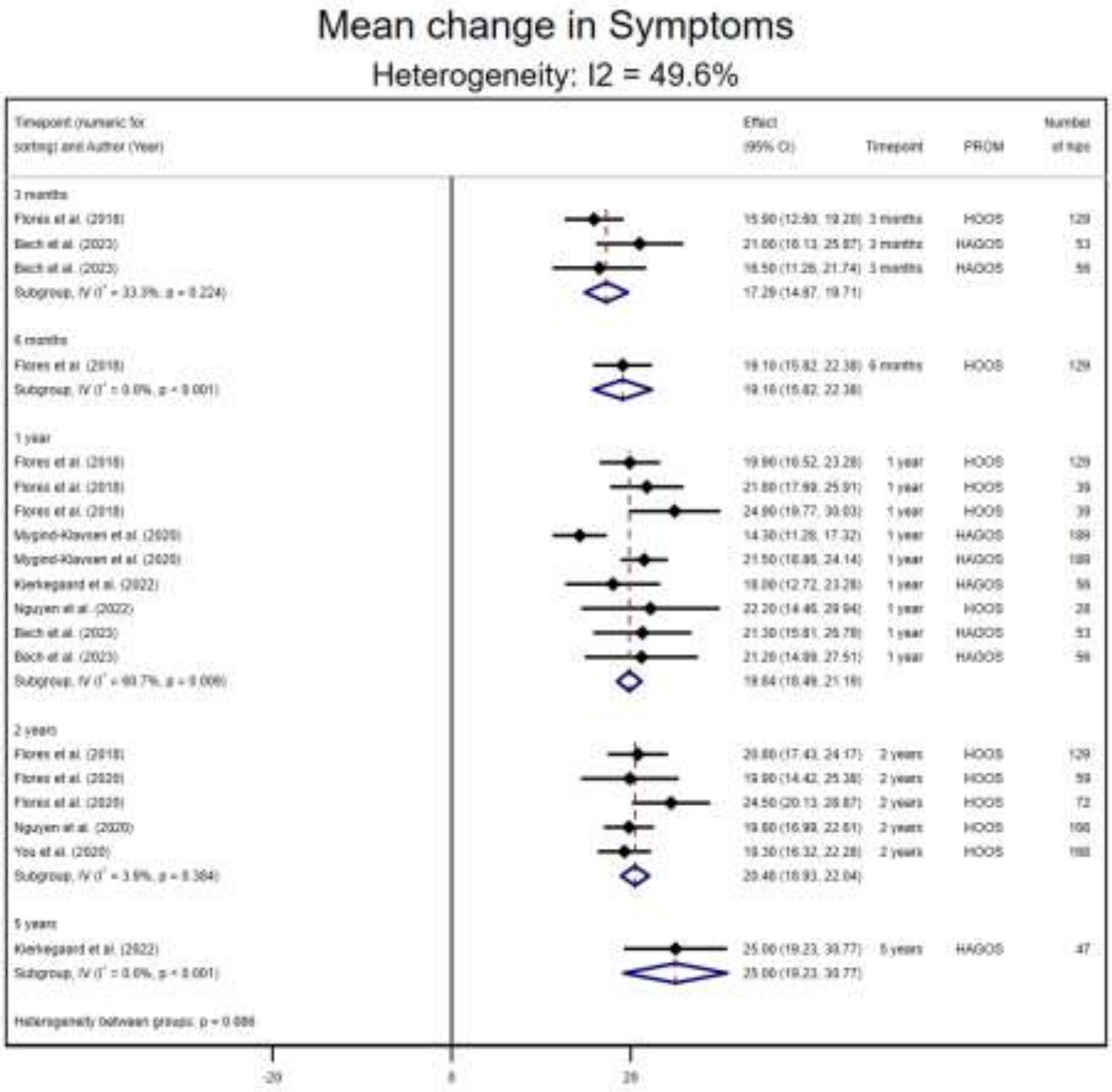

Supplementary Figure 24. Change in the composite Total score from baseline to each follow-up, after primary hip arthroscopy in patients with femoroacetabular impingement.

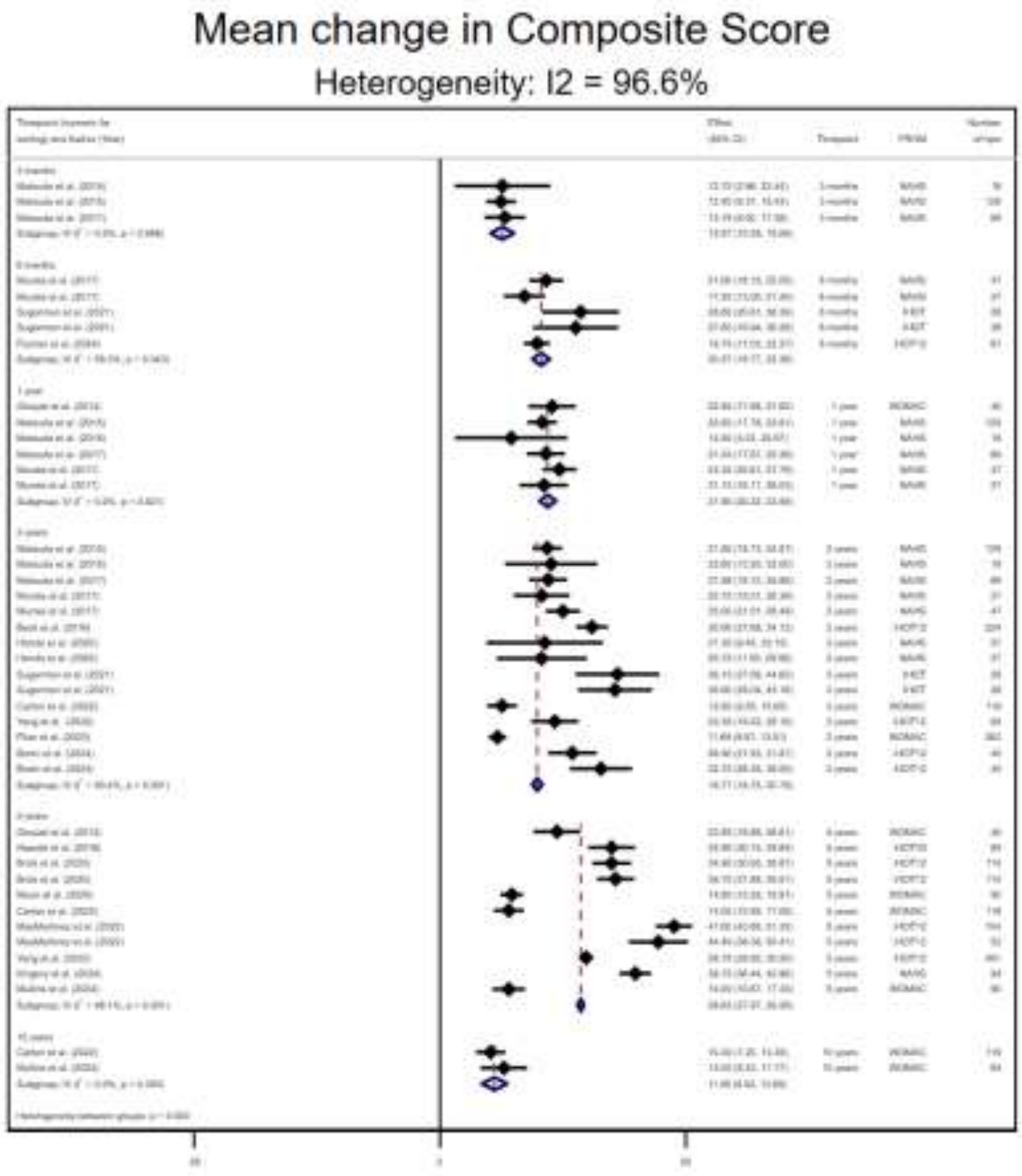

Benefits across hip-related outcomes, sub-analysis of Capsular Repair or no Capsular Repair.

Supplementary Figure 25. Change in Pain subscales from baseline to each follow-up, after primary hip arthroscopy in patients with femoroacetabular impingement. Capsular repair or no capsular repair.

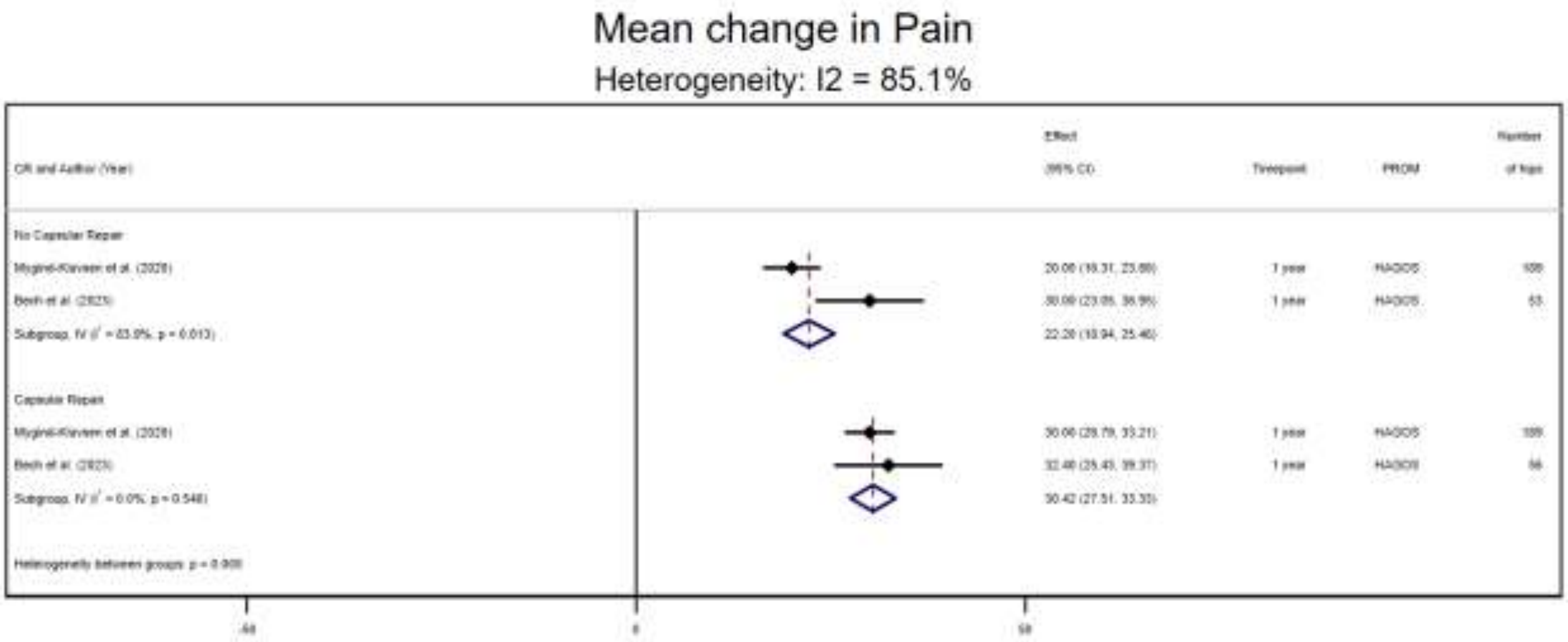

Supplementary Figure 26. Mean change in the Activities of Daily Living subscales from baseline to each follow-up, after primary hip arthroscopy in patients with femoroacetabular impingement. Capsular repair or no capsular repair.

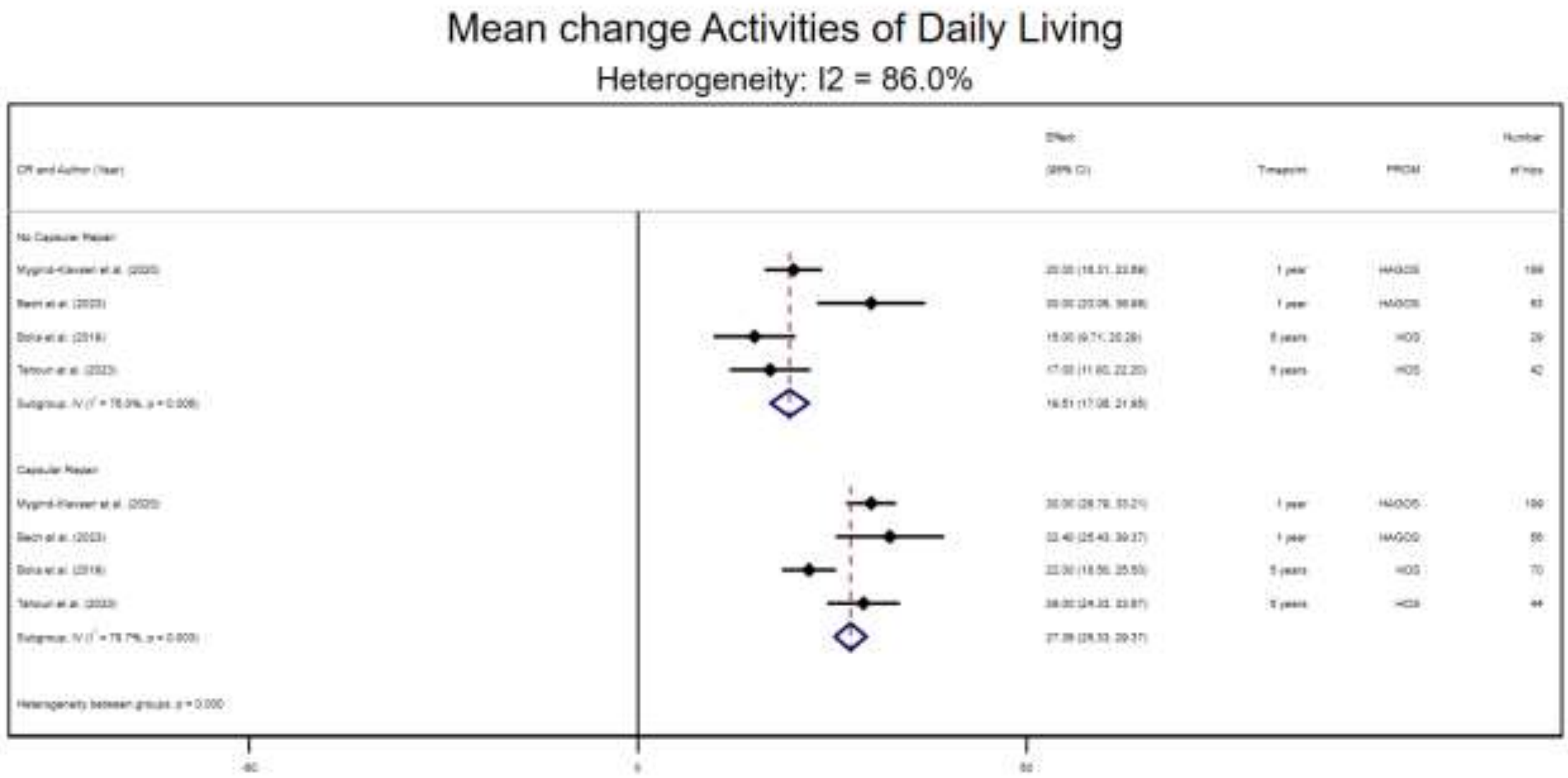

Supplementary Figure 27. Mean change in the Sports Participation subscales from baseline to each follow-up, after primary hip arthroscopy in patients with femoroacetabular impingement. Capsular repair or no capsular repair.

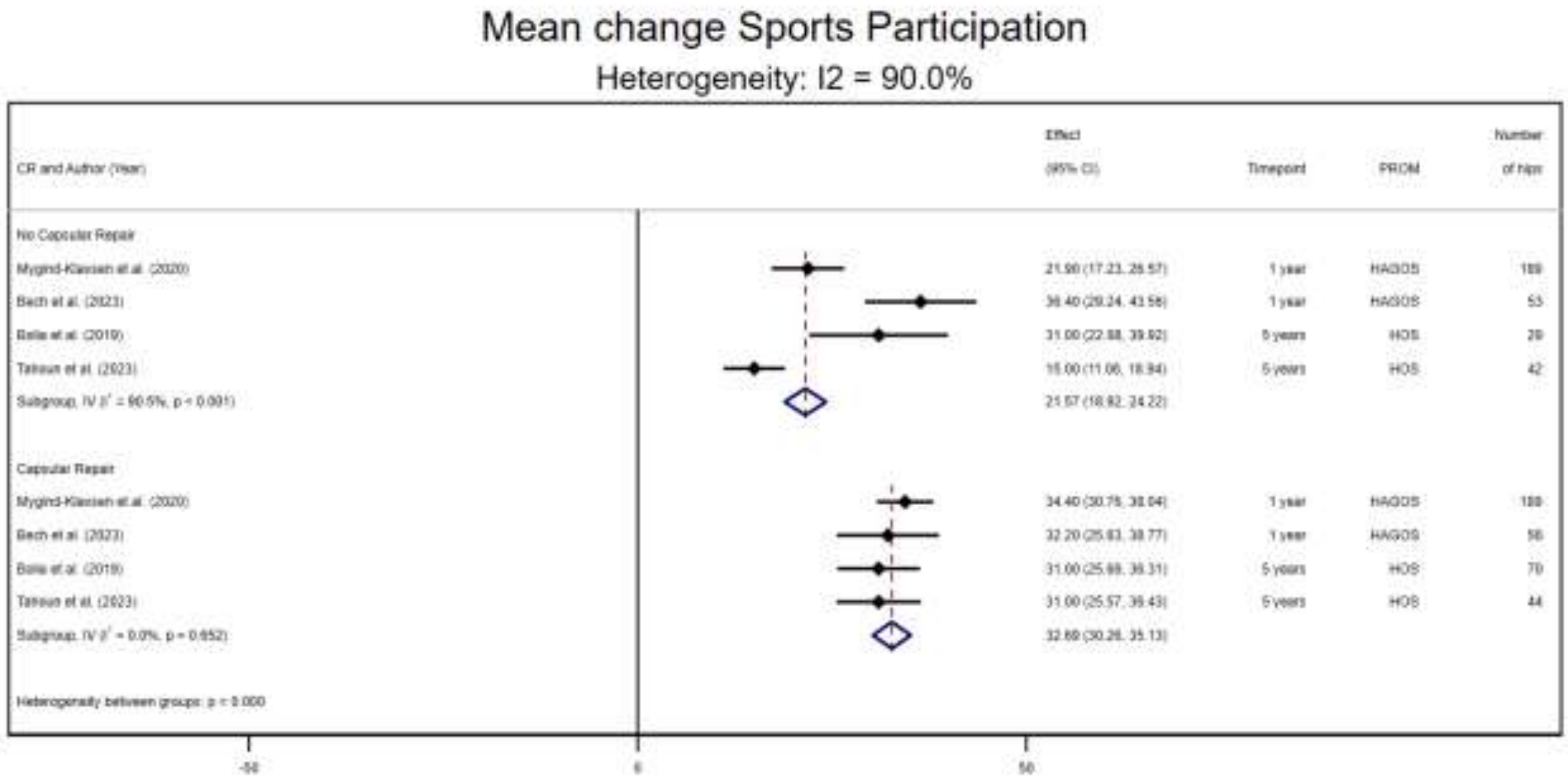

Supplementary Figure 28. Mean change in the Quality-of-Life subscales from baseline to each follow-up, after primary hip arthroscopy in patients with femoroacetabular impingement. Comparing Capsular repair or no capsular repair.

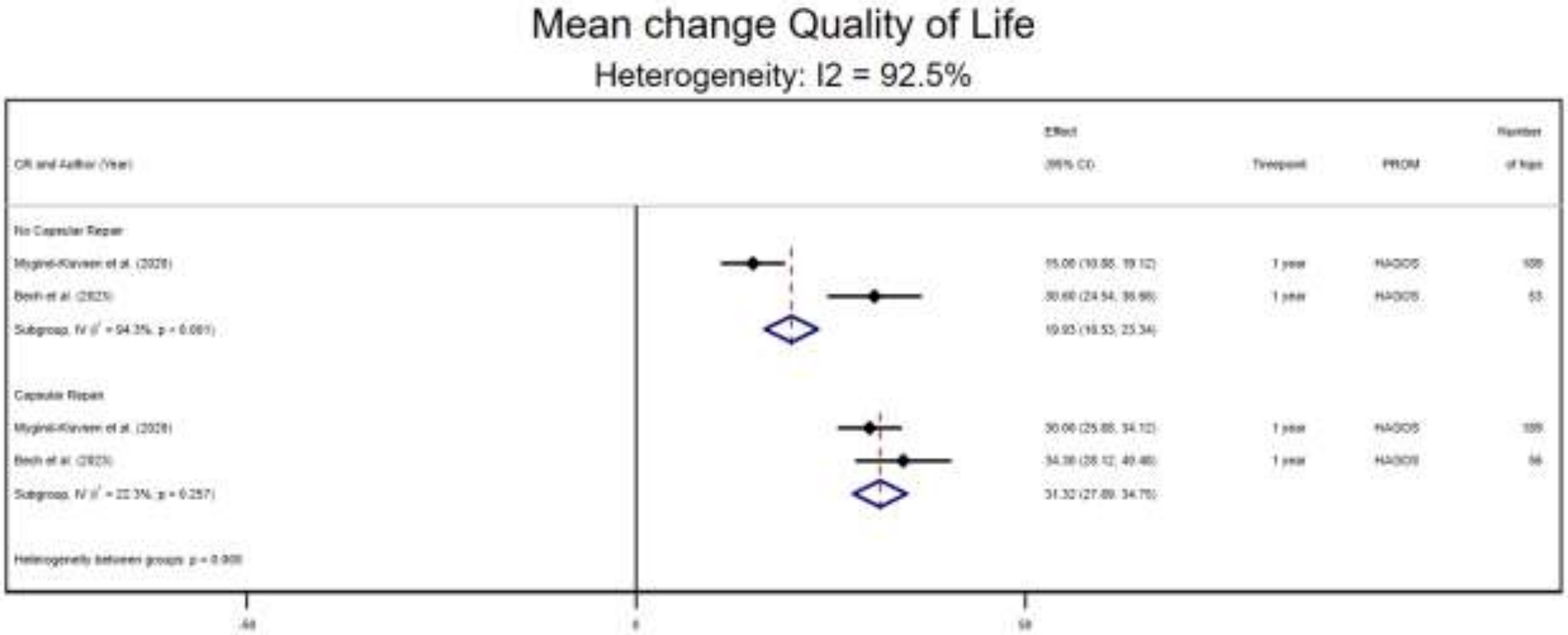

Supplementary Figure 29. Mean change in the Symptoms subscales from baseline to each follow-up, after primary hip arthroscopy in patients with femoroacetabular impingement. Comparing Capsular repair or no capsular repair.

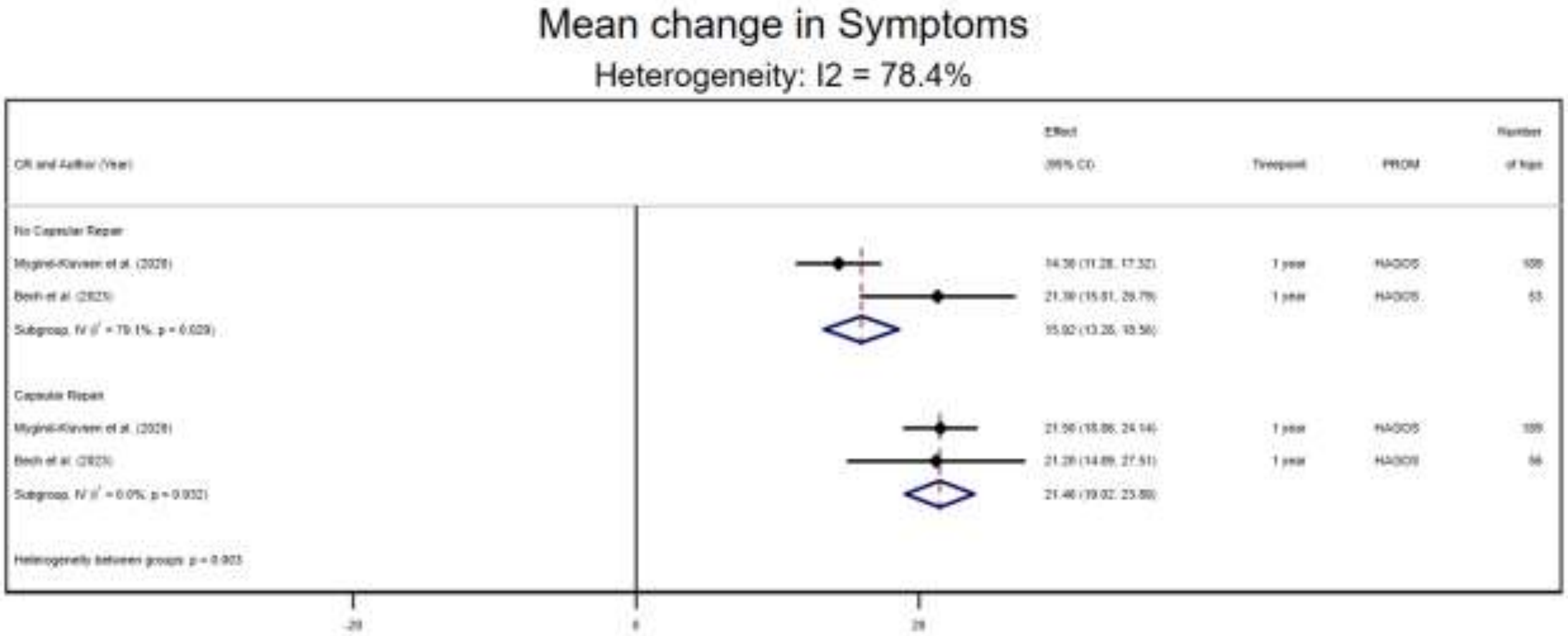

Supplementary Figure 30. Mean change in the composite Total scores from baseline to each follow-up, after primary hip arthroscopy in patients with femoroacetabular impingement. Comparing Capsular repair or no capsular repair.

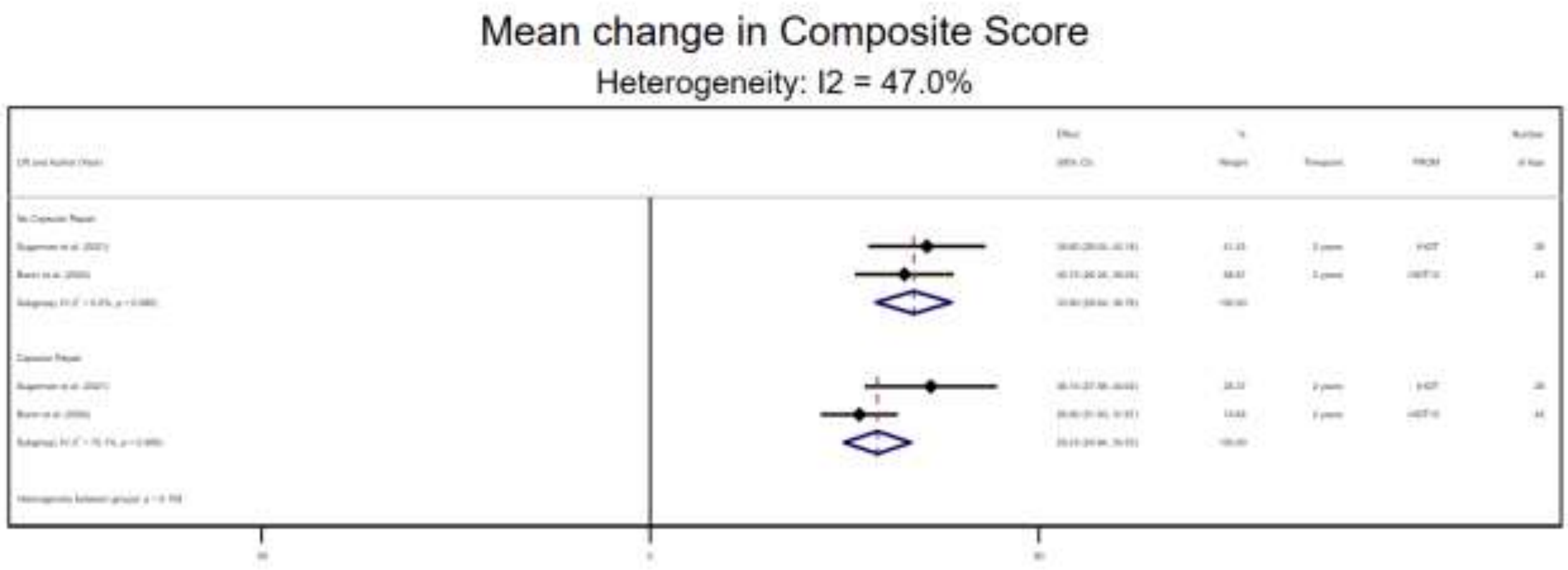

Funnel plots.

Supplementary Figure 31. Funnel plot of studies reporting incidence of conversion to total hip arthroplasty and revision arthroscopy following hip arthroscopy.

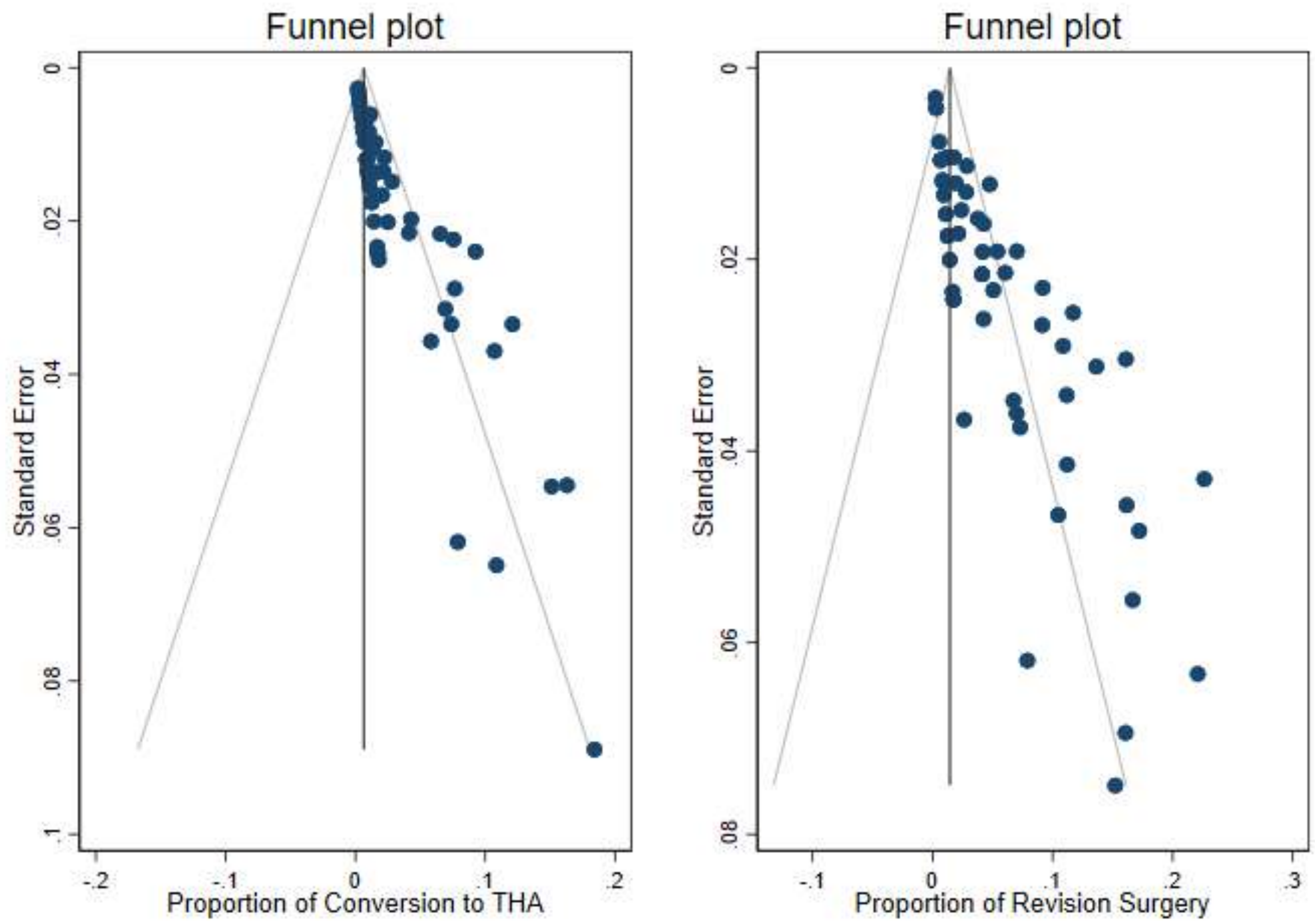

Supplementary Figure 32. Funnel plot of studies reporting patient-reported outcome measures.

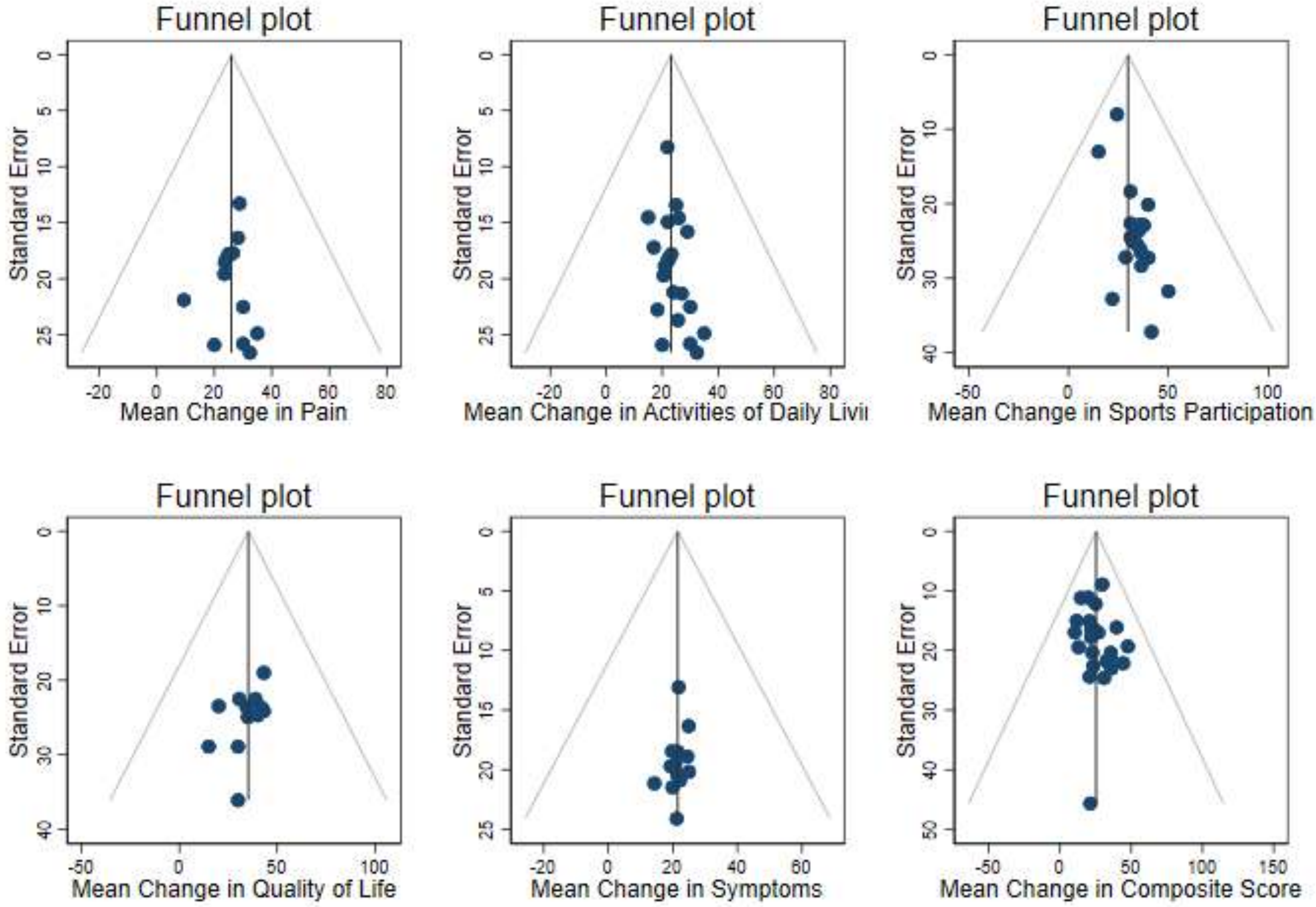

Supplement: Supplementary file 1 [file ARS2-8-e70036-s001.pdf]
